# Trajectories of Suicidal Risk Impact Mood Regulation Differently in Patients With a Diagnosis of Bipolar Disorder

**DOI:** 10.1111/acps.70094

**Published:** 2026-04-02

**Authors:** Abigail Ortiz, Ramzi Halabi, Daniel Blumberger, Christina Gonzalez‐Torres, Arend Hintze, Muhammad I. Husain, Mirkamal Tolend, Juveria Zaheer, Martin Alda, Benoit H. Mulsant

**Affiliations:** ^1^ Department of Psychiatry, Faculty of Medicine UT Southwestern Medical Center Dallas Texas USA; ^2^ Department of Psychiatry, Temerty Faculty of Medicine University of Toronto Toronto Ontario Canada; ^3^ Centre for Addiction and Mental Health (CAMH) Campbell Family Research Institute Toronto Ontario Canada; ^4^ Department of Psychiatry Western University Schulich School of Medicine & Dentistry London Ontario Canada; ^5^ Department of MicroData Analytics Dalarna University Falun Sweden; ^6^ National Institute of Mental Health Klecany Czech Republic; ^7^ Department of Psychiatry Dalhousie University Halifax Nova Scotia Canada; ^8^ The Royal Ottawa Hospital Ottawa Ontario Canada

**Keywords:** bipolar disorder, mood regulation, suicidal behavior, time‐series analysis

## Abstract

**Background:**

Bipolar disorder (BD) carries a suicide risk 20 times higher than the general population, with up to 60% of patients attempting suicide. Current interventions have failed to reduce its incidence; static factors have shown limited predictive utility. Emerging evidence suggests dynamic monitoring approaches may offer complementary value. This study examined whether quantifiable differences in mood regulation patterns exist across the suicidality continuum among patients diagnosed with BD.

**Method:**

We analyzed daily self‐reported mood, anxiety, and energy levels from 164 participants recruited from two Canadian academic hospitals (April 2021–August 2024). Participants were stratified into six groups based on suicide attempt history, current polarity, and active suicidality status. Using time‐series analysis, we computed autocorrelation and cross‐correlation functions to examine temporal relationships within and between variables across 1–7 day lags. Data comprised 64,351 valid observations over 461.5 ± 236.6 days of follow‐up.

**Results:**

Participants with the highest suicide risk (previous attempt, in a current depressive episode with active suicidality) demonstrated significantly higher day‐to‐day autocorrelation compared to the lowest‐risk participants (no prior attempts and currently euthymic) for mood (0.53 vs. 0.29, *p* = 0.01), energy (0.52 vs. 0.23, *p* = 0.02), and anxiety series (0.55 vs. 0.32, *p* = 0.04). Cross‐correlation analysis revealed mood‐energy decoupling during active suicidality; as well as a stronger negative mood‐anxiety correlation in those with a prior attempt, even during euthymia.

**Conclusion:**

Higher autocorrelation patterns are indicative of a pathologically stable mood regulation in high‐risk individuals, potentially serving as dynamic biomarkers for suicide risk stratification and targeted intervention development. Our findings demonstrate that a more aggressive approach to treating comorbid anxiety may be essential for reducing the risk of future attempts. They also challenge traditional conceptualizations equating euthymia with the absence of suicide risk, suggesting neurobiological vulnerability despite symptomatic remission.

## Key Points

- What is known about this topic?
  ○ Bipolar disorder carries a suicide risk approximately 20 times higher than the general population.
  ○ Known clinical risk factors include a history of prior attempts, depressive polarity, mixed features, comorbid anxiety disorders; however, these factors capture population‐level risk.
  ○ Mood variability, rather than mood severity alone, captures clinically meaningful information about illness course in bipolar disorder.
- What this paper adds to our knowledge
  ○ Individuals at highest suicide risk (prior attempt, current depressive episode, active suicidality) exhibit significantly elevated day‐to‐day autocorrelation in mood, energy, and anxiety compared to lowest‐risk individuals.
  ○ Participants with a history of suicide attempts show persistent dysregulation between mood and anxiety levels, even during euthymia, suggesting that suicide risk should not be assumed absent during periods of remission.
  ○ These findings establish quantifiable temporal biomarkers of suicide risk detectable from routine self‐report data, with direct implications for passive monitoring systems.

## 1 Introduction

Bipolar disorder (BD) is a mood disorder with an estimated suicide risk 20 times higher than the general population [1, 2, 3]. Up to 60% of patients diagnosed with BD attempt suicide at least once in their lifetime, and 19% ultimately end their lives by suicide [4], with the highest risk during depressive [5, 6], and mixed episodes [7, 8]. The risk is even higher in those with a family history of suicide [6, 9, 10, 11], and a history of a previous attempt [12, 13, 14, 15].

While several interventions for relapse and suicide prevention have been developed [16, 17], these strategies have not decreased the incidence of relapse or suicide [6, 18]. In parallel, while the persistent high incidence of suicide in BD is multifactorial, traditional approaches relying primarily on cross‐sectional assessments [19] or static risk factors (e.g., age, sex) have shown limited predictive utility [18, 20]. Moreover, even though the highest‐risk window for suicide attempts is following a hospital discharge [21], only 1% of studies have observed participants during this window [18], and usually for no longer than a month [22]. Emerging evidence suggests dynamic monitoring approaches may offer complementary value [23, 24], for instance, by capturing the rapid fluctuations in mood [5, 25], anxiety [6, 26], sleep [7, 27], and other behaviors (e.g., impulsivity [28, 29]) that precede suicide attempts.

Recent studies using ecologic momentary assessment (EMA) in patients diagnosed with a mood disorder have shown that suicidal ideation changes over time (i.e., hours or days) [19, 23, 24, 30]. These changes are related to interpersonal variables (e.g., hopelessness) [31] and anxiety [32, 33], with insomnia [34, 35, 36] as a moderator of this relationship [37, 38, 39, 40]. In BD, recent studies using EMA have shown temporal associations between changes in energy, accurately predating increases in low mood [41], and changes in negative affect predating an increase in suicidal ideation [42].

Furthermore, while some studies suggest a differential modulatory effect of anxiety on suicidal ideation that varies significantly between individuals with and without prior suicide attempts [43], there are substantial gaps in understanding the temporal relationship between mood regulation, anxiety, and energy levels in patients diagnosed with BD with and without prior suicide attempts.

The relationship between mood variability, anxiety, and energy levels can be understood within the context of mood regulation. We have previously described mood regulation as a complex and poorly understood process, which can be conceived as a “buffer” system that allows flexible and adaptive responses to changing conditions [44]. From a dynamic perspective, the properties that enable this adaptability are considered “complex.” Nonlinear methods provide new tools for quantifying, modeling, and predicting the behavior of complex systems [45, 46]. Time‐series analysis is a type of nonlinear method employed to study a collection of observations made sequentially in time [47], which identifies the degree of order (or regularity) in what otherwise may be viewed as random or disordered serial data [48, 49]. Typically, highly correlated series may be indicative of illness, as in cardiovascular [50], metabolic diseases [51], and psychiatric diseases [52, 53, 54, 55]. Our previous work has described highly correlated series (i.e., a “pathologically stable” or organized mood [56]) in euthymic participants diagnosed with BD and their unaffected first‐degree relatives [57, 58], as well as in those patients with a history of antidepressant‐induced mania [59].

The aim of this study was to examine intra‐individual variability in mood regulation by analyzing daily self‐reported mood, anxiety, and energy levels among participants diagnosed with BD; stratified by (i) presence or absence of a prior suicide attempt; (ii) current polarity (i.e., euthymic or depressive); and (iii) presence or absence of current (i.e., active) suicidality. We hypothesized that quantifiable differences in mood regulation parameters would emerge across the suicidality continuum. Specifically, we hypothesized that participants categorized as high‐risk (i.e., those with a prior attempt and currently in a depressive episode, with suicidal ideation) would demonstrate distinctive patterns of mood regulation compared to those classified as low risk (i.e., those without prior suicide attempts and currently euthymic).

### 1.1 Aims of the Study

This study aimed to examine whether quantifiable differences in mood regulation patterns exist across the suicidality continuum in participants with bipolar disorder. Using time‐series analysis of daily self‐reported affect, we computed autocorrelation and cross‐correlation across participants stratified by suicide attempt history, current polarity, and active suicidality status. We hypothesized that participants at higher suicide risk would demonstrate distinct patterns of mood regulation compared to lower‐risk participants.

## 2 Methods

### 2.1 Participants

The participants whose data is analyzed in this paper are part of an ongoing research study, which we previously described in detail [60]. Briefly, the study was conducted at two Canadian academic hospitals: the Centre for Addiction and Mental Health (CAMH) in Toronto, Ontario, and the Queen Elizabeth II Health Sciences Centre in Halifax, Nova Scotia. Fully informed by the Privacy Office at both institutions, the Research Ethics Board (REB) approval (# 059‐2019) was obtained at each of the abovementioned academic centers, in accordance with the Declaration of Helsinki. Inclusion criteria were participants aged 18 and over, with a primary diagnosis of BD type I or II. Exclusion criteria were participants with a comorbid personality disorder, comorbid substance abuse or dependence, and a diagnosis of a mood disorder secondary to another general medical cause. Recruitment for this cohort occurred between April 2021 and August 2024 from outpatient clinics specialized in BD and from inpatient units a week after discharge following a suicide attempt. Upon discharge, participants were followed weekly by a study psychiatrist for 6 months. Participants received treatment according to standard local practices.

### 2.2 Data Description

#### 2.2.1 Baseline Assessments

Primary diagnoses were made according to the Diagnostic and Statistical Manual of Mental Disorders 5 (DSM‐5) [61] confirmed by the Structured Clinical Interview for DSM‐5 (SCID‐5) [62]. After all procedures had been fully explained and written consent was obtained, a comprehensive baseline assessment was completed: participants provided information regarding their sociodemographic characteristics, clinical course, cardiovascular status, chronotype, and pharmacotherapy. Race, ethnicity, sex at birth, and gender identity were self‐reported. To determine the polarity of each participant (i.e., euthymia, depression, or (hypo)mania), the Young Mania Rating Scale (YMRS) [63] and the Montgomery‐Asberg Depression Rating Scale (MADRS) [64] were administered by a trained rater upon entry to the study. Euthymia was defined as YMRS ≤ 10 and MADRS ≤ 10 for a minimum of 4 weeks; depressive polarity was defined as MADRS ≥ 10 for 2 weeks, and (hypo)manic polarity was defined as YMRS ≥ 10 for a week. We chose our cutoffs to reflect real‐world data, thereby improving the generalizability of our results and their applicability in non‐research settings, and based our decisions on cutoffs in influential papers [65, 66, 67, 68, 69].

#### 2.2.2 Data Collection

Building on our method developed to analyze mood regulation using time‐series analysis [57, 58, 59], mood, anxiety, and energy levels were monitored daily using an electronic visual analog scale (e‐VAS). The scale ranged from 0 to 100, with 50 representing the participant's “usual self.” Participants provided their daily ratings through REDCap electronic data capture tools hosted at CAMH and the Queen Elizabeth II Health Sciences Centre [70]. The e‐VAS featured one‐point intervals, allowing the collection of densely‐sampled, continuous data. To ensure data completeness, participants were required to rate all three e‐VAS components (i.e., mood, anxiety, and energy) before submitting their daily entries. Email reminders were sent to participants after three consecutive days of missing entries. In addition, MADRS and YMRS weekly scores were collected weekly by a study psychiatrist to monitor symptom severity over the study course, and these scores were used for week‐by‐week labeling.

### 2.3 Suicidality Risk Stratification

Participants were stratified into groups based on their risk of suicidality and clinical status, as determined by weekly MADRS scores. As above, we defined a depressive episode as MADRS scores ≥ 10 for two or more consecutive weeks.

**FIGURE 1:**
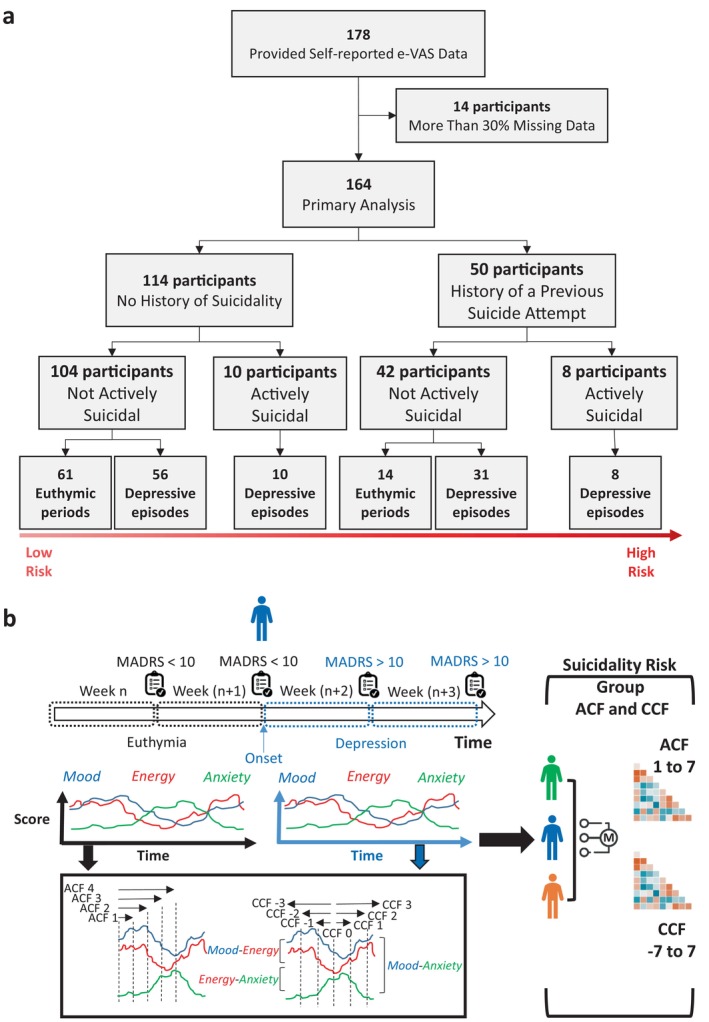
Study design. Illustration of (a) the study design and suicidality risk group stratification, and (b) data pipeline from data collection to ACF and CCF matrix computation at an individual and group level.

The classification of suicidality risk followed a hierarchical three‐level stratification approach (Figure 1a). Initially, participants were categorized based on prior history of suicidality (presence versus absence), with each resultant group subsequently divided according to current suicidality status (active versus inactive). The final stratification integrated the current affective state assessment (euthymic versus depressed), yielding six distinct clinical groups (as all those with active suicidal ideation were depressed) that captured the temporal dynamics of suicidality risk alongside clinical characteristics.

As participants changed in their clinical presentation (e.g., transitioned from a depressive episode to euthymia), this resulted in non‐exclusive group allocation across the assessment period. For instance, a participant might contribute data to the group characterized by a history of a prior attempt, currently in a depressive episode, and reporting active suicidality, for 4 months, but following clinical improvement, subsequently contribute data to a different group (e.g., history of a prior attempt, currently euthymic, and not reporting suicidality) for an additional 2 months. This methodological approach acknowledges the temporal dynamics of suicidality risk factors, capturing the fluidity of clinical presentations rather than imposing static categorizations that fail to reflect the inherent variability of psychopathological phenomena.

### 2.4 Data Assumptions

The analyses were based on two key assumptions. The first assumption involved selecting an appropriate model for the data. Given the presence of noise in the dataset, we employed a basic model capable of capturing fundamental patterns, such as an autoregressive process AR (1), which examines the relationship between consecutive data observations. The second assumption was made about the temporal stability of the data. While perfect consistency over time is ideal, real‐world data exhibits variability. To address this, we treated our data as weakly stationary, requiring only that the first two moments of the data (mean and variance) remain relatively constant over time [57]. This approach allowed us to account for natural fluctuations in the data while ensuring a stable foundation for the correlation analysis. To verify stationarity, we employed the augmented Dickey‐Fuller test [71].

### 2.5 Data Selection and Preprocessing

Building on our previous findings that self‐reported e‐VAS data is missing not at random (MNAR) [72], we applied the *K*‐nearest neighbors (KNN) algorithm for imputing missing observations [73]. This approach identifies the k‐nearest neighbors to a missing value using a specified distance metric and imputes the value by calculating the median of these neighbors. Participants with more than 30% missing data (*n* = 14) were excluded from the analysis.

### 2.6 Data Analysis

After confirming stationarity of the time series and removing spurious interdependencies through detrending, we performed a two‐dimensional correlation analysis across clinical subgroups: (i) Autocorrelation Function (ACF) to explore temporal relationships within each e‐VAS variable across lags of 1–7 days (e.g., comparing mood today with mood on previous days across the six suicidality risk groups); and (ii) cross‐correlation function (CCF) to explore temporal relationships between pairs of e‐VAS variables across lags ranging from −7 to 7 days (e.g., comparing mood today with anxiety yesterday) across the six suicidality risk groups. The ACF quantifies how closely a time series resembles itself over specific time lags, with higher values indicating stronger correlations between observations separated by those lags. Similarly, the CCF measures the degree of association (i.e., coupling) between two time series across lags, where higher values indicate stronger correlations between variables.

### 2.7 Statistical Analysis

To compare both ACF and CCF features across the six suicidality risk groups, we computed the median and inter‐quartile range (IQR) of ACF and CCF coefficients for each lag, as well as the differences in those coefficients across the six groups using Fisher's *Z*‐transformation [74], which standardizes correlation coefficients into *Z*‐scores and quantifies the level of difference between the coefficients across groups. Each data segment (e.g., weeks in a depressive episode) was treated independently, as it represented a distinct and non‐overlapping clinical polarity. This segment‐wise approach allowed the extraction and comparison of group‐specific ACF and CCF features. Confidence intervals for ACF and CCF coefficients' group medians were estimated using bootstrap resampling with 1000 iterations, and pairwise comparisons between groups were corrected for multiple testing using the Bonferroni method. The median *Z*‐statistics and corrected *p* values, as well as the bootstrapped confidence intervals for group medians, were systematically reported for both ACF and CCF analyses. Statistically, we controlled for age, sex, and treatment type. All statistical computations were performed using Python 3.11. See Figure 1b for a detailed depiction of the data analysis pipeline.

### 2.8 Supplementary Analyses

To ensure the robustness of our findings, we conducted a series of supplementary association and sensitivity analyses regarding data completeness (cross‐sectional and longitudinal) and imputation effect on time‐series dynamics within and across suicide risk groups (see Table S1). To assess the sensitivity of the temporal dynamics to the data completion strategy, we compared the ACF features derived from raw e‐VAS data with those obtained using Multiple Imputation by Chained Equations (MICE) (see Figures S1–S3). We then evaluated whether data completeness was associated with clinical status by comparing the missing data ratios (MDR) across the six risk groups and assessing the correlation between MDR and e‐VAS dynamics (i.e., ACF and CCF) (see Figures S4–S12). Finally, we characterized the temporal structure of missing data gaps by analyzing the autocorrelation of a binary missingness indicator and generating longitudinal MDR profiles using a sliding‐window approach on a normalized 0%–100% follow‐up axis (see Figures S13 and S14). Detailed descriptions of these procedures are provided in the Supporting Information: Methods.

## 3 Results

Of a total of 178 participants diagnosed with BD, 160 were recruited from outpatient clinics, and 18 from inpatient units following a suicide attempt. In this analysis, 164 participants were included (146 outpatients and 18 inpatients); the remaining 14 participants did not provide sufficient data for a reliable time‐series analysis (i.e., they had more than 30% missing data). As described in Section 2, we performed an additional sensitivity analysis (see Supporting Information) to corroborate that our results were not affected by data completeness.

Over half of the participants (*n* = 104; 63.3%) had no history of suicide attempts nor were they actively suicidal; while eight participants (4.8%) had a history of one or more suicide attempts and were actively suicidal. No statistically significant differences were found across groups for demographic or clinical characteristics. Data included a mean (SD) of 430.1 (242.2) person‐days of daily e‐VAS and 46.2 (34.8) person‐days of valid weekly MADRS and YMRS, with 17,495 missing observations (79.22% compliance) across 461.5 ± 236.6 (mean ± SD) days. A detailed description of the participants' sociodemographic and clinical features is presented in Table 1.

**TABLE 1:** Demographic and clinical characteristics of the sample.

Characteristics	All participants (*N* = 164)
Age, mean (SD)	38.3 (12.3)
Sex assigned at birth, *n* (%)	
Male	61 (37.2)
Female	103 (62.8)
Gender, *n* (%)	
Man	55 (33.5)
Woman	88 (53.7)
Queer/gender non‐conforming	4 (2.4)
Prefer not to disclose	17 (10.4)
Education, *n* (%)	
Completed high school or less	32 (19.5)
Completed college	7 (4.3)
Completed university	125 (76.2)
Marital status, *n* (%)	
Single	84 (51.2)
Married	57 (34.8)
Divorced	21 (12.8)
Widowed	2 (1.2)
Socioeconomic status, *n* (%)	
Work full‐time	85 (51.8)
Work part‐time	22 (13.4)
Social assistance or disabled	18 (11.0)
Student	11 (6.7)
Unemployed	22 (13.4)
Retired	6 (3.7)
Primary diagnosis, *n* (%)	
Bipolar disorder I	105 (64.0)
Bipolar disorder II	59 (36.0)
Rapid cycling, *n* (%)	36 (22.0)
Clinical status at study entry, *n* (%)	
Euthymic	98 (59.8)
In a depressive episode	62 (37.8)
In a hypomanic episode	2 (1.2)
In a mixed episode	2 (1.2)
MADRS baseline score per clinical status at study entry, mean (SD)	
Euthymic	3.0 (3.6)
In a depressive episode	21.6 (8.9)
In a manic or hypomanic episode	1.0 (1.4)
In a mixed episode	23.0 (1.4)
YMRS baseline score per clinical status at study entry, mean (SD)	
Euthymic	0.4 (1.4)
In a depressive episode	1.6 (2.4)
In a manic or hypomanic episode	11.0 (1.4)
In a mixed episode	13.5 (0.7)
MADRS score per suicide risk group, mean (SD)	
Euthymic, no suicide history	8.4 (3.6)
Euthymic, suicide history	11.4 (4.8)
Depressed, no suicide history, not actively suicidal	7.2 (3.3)
Depressed, suicide history, not actively suicidal	9.5 (4.2)
Depressed, no suicide history, actively suicidal	16.1 (5.9)
Depressed, suicide history, actively suicidal	18.3 (7.0)
YMRS score per suicide risk group, mean (SD)	
Euthymic, no suicide history	2.1 (1.8)
Euthymic, suicide history	2.1 (2.0)
Depressed, no suicide history, not actively suicidal	2.2 (1.8)
Depressed, suicide history, not actively suicidal	2.3 (1.9)
Depressed, no suicide history, actively suicidal	1.3 (1.6)
Depressed, suicide history, actively suicidal	1.6 (2.3)
Daily self‐reported e‐VAS observations, mean (SD)	430.1 (242.2) person‐days
Weekly self‐reported MADRS/YMRS observations, mean (SD)	46.2 (34.8) person‐weeks
Mean (SD) episode duration (days)	105.1 (181.3)
Suicidality risk, *n* (%)	
No history of suicidality, no active suicidality	104 (63.3)
History of suicidality, no active suicidality	42 (26.1)
No history of suicidality, active suicidality	10 (5.9)
History of suicidality, active suicidality	8 (4.8)
Pharmacotherapy at study entry, *n* (%)	
On no treatment at study entry, *n* (%)	2 (1.2)
Monotherapy	38 (23.2)
Lithium	5 (3.1)
Anticonvulsant	17 (10.3)
Atypical antipsychotic	16 (9.7)
Combination treatment	124 (75.6)
Lithium + anticonvulsant	9 (5.5)
Lithium + atypical antipsychotic	24 (14.6)
Anticonvulsant + atypical antipsychotic	34 (20.7)
Lithium + anticonvulsant + atypical antipsychotic	9 (5.5)
Any of the above + antidepressant	48 (29.2)

### 3.1 Autocorrelation Analysis

**FIGURE 2:**
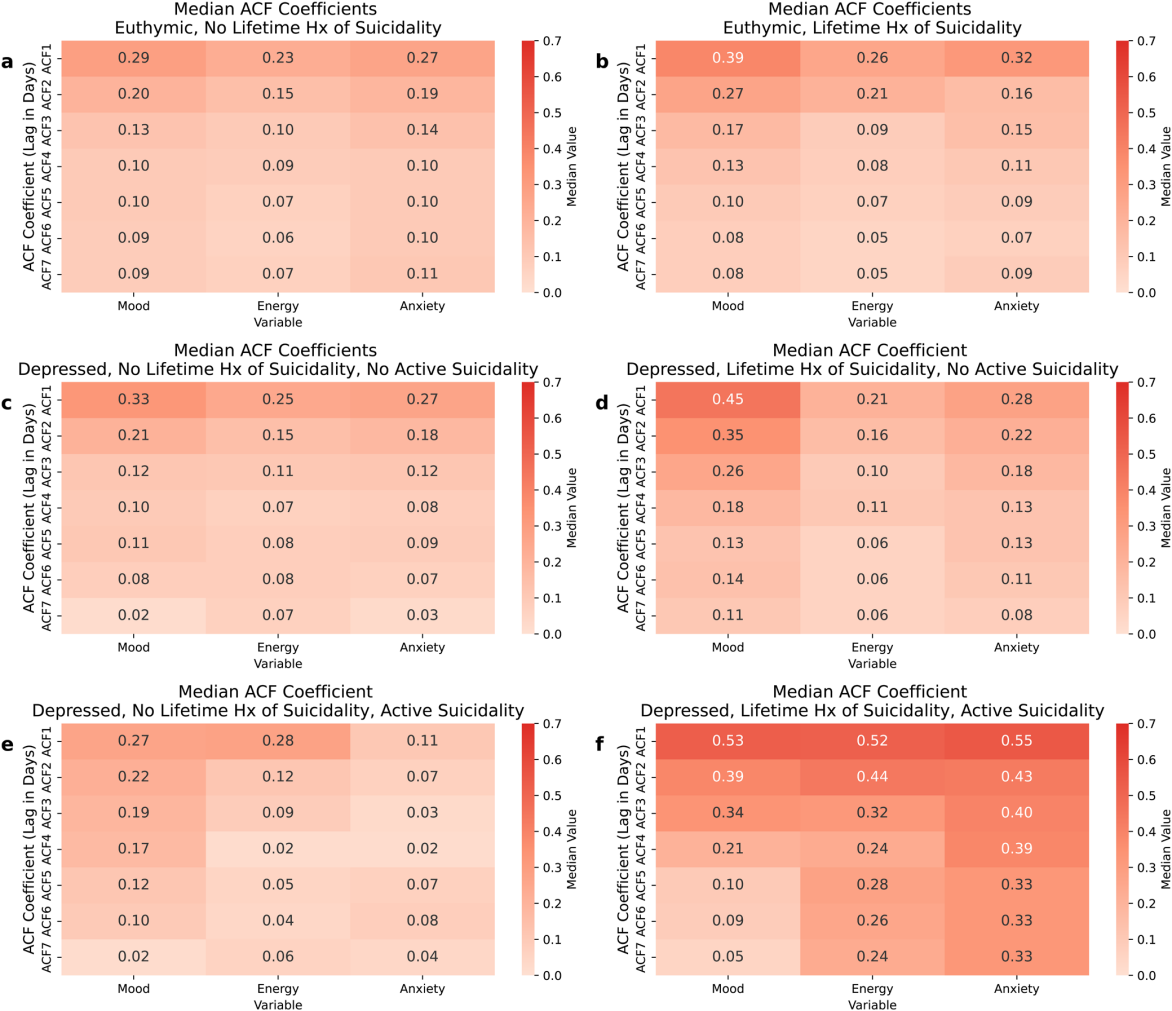
Autocorrelation analysis matrices (Lags 1–7) of mood, energy, and anxiety time series, stratified by increasing risk of suicidality. Autocorrelation analysis matrices in: (A) currently euthymic with no prior suicide attempts; (b) currently euthymic, history of suicide attempts; (c) in a depressive episode, not actively suicidal, no prior suicide attempts; (d) in a depressive episode, not actively suicidal, with a history of suicide attempts; (e) in a depressive episode, actively suicidal, with no prior suicide attempts; and (f) in a depressive episode, actively suicidal, with prior suicidal attempts.

The highest autocorrelation coefficients for participants across all groups were observed at Lag 1 (i.e., day‐to‐day self‐similarity, Figure 2), as we have previously reported [57].

During euthymia, participants with a prior attempt showed higher day‐to‐day autocorrelation in the anxiety series (0.31 (0.26–0.38)), compared to those who were currently depressed, not suicidal, and without a history of prior attempt (0.11 (−0.04 to 0.58), *p* = 0.03). For mood and energy, participants with a prior attempt exhibited higher autocorrelation at Lag 1 for mood: 0.39 (0.31–0.45) and energy: 0.26 (0.19–0.34), compared to those without a history of attempts (mood: 0.29 (0.25–0.38); energy: 0.23 (0.20–0.29)). Differences were not statistically significant (mood, Fisher's *Z*‐statistic = −9.56, *p* = 0.17; energy, Fisher's *Z*‐statistic = −0.16, *p* = 0.61).

During depressive episodes, participants who were actively suicidal and had a history of attempt (i.e., highest‐risk group), showed the highest day‐to‐day autocorrelation for all series: mood, 0.53 (0.53–0.59); energy, 0.52 (0.27–0.56); anxiety, 0.55 (0.40–0.60), significantly higher compared to those who were euthymic and had no prior attempts (i.e., lowest‐risk group): mood, 0.29 (0.25–0.38), *p* = 0.01; energy, 0.23 (0.20–0.29), *p* = 0.02, anxiety, 0.32 (0.26–0.38), *p* = 0.04. In parallel, the highest‐risk group showed higher day‐to‐day autocorrelation in the energy series compared to those who were euthymic and had had prior attempts (0.26 (0.19–0.34), *p* = 0.03). Also, the highest‐risk group showed higher day‐to‐day autocorrelation in the anxiety series compared to those who were depressed but had no history of attempts and were not actively suicidal (0.27 (0.19–0.3), *p* = 0.03). Similarly, participants with a prior attempt who were currently depressed but not suicidal showed higher day‐to‐day autocorrelation in the mood series (0.45 (0.22 = 0.59)) compared to those who were euthymic and without prior attempts (0.29 (0.25–0.38); *p* = 0.03). See Figure 2 for the detailed representation of ACF coefficients across classes, and Table S1 for the comparative autocorrelation statistics at all lags (1–7) across suicidality risk groups.

### 3.2 Cross‐Correlation Analysis

#### 3.2.1 Cross‐Correlation Between Mood and Energy (Lags −7 to 7)

The highest positive cross‐correlation between mood and energy across risk groups was observed at Lag 0 (e.g., between a participant's mood and energy on the same day).

Participants who were currently depressed, not actively suicidal, and without prior attempts showed a higher cross‐correlation between mood‐energy (0.53 (0.49–0.63)) compared to those who were currently euthymic, with or without a prior attempt (0.44 (0.42–0.53); *p* = 0.04; 0.45 (0.38–0.50); *p* = 0.03).

Moreover, participants who were depressed, actively suicidal, but without prior attempts showed significantly lower mood‐energy cross‐correlation (0.33 (0.08–0.6)) than those who were depressed, were not actively suicidal, but had had attempts (0.51 (0.44–0.55); *p* = 0.04); and also significantly lower than those who were depressed, were not actively suicidal, and without prior attempts (0.53 (0.49–0.63); *p* = 0.04). Cross‐correlation coefficients between mood and energy beyond a single‐day lag were negligible and did not differ statistically between groups.

#### 3.2.2 Cross‐Correlation Between Mood and Anxiety Series (Lags −7 to 7)

Similarly to the mood‐energy dynamics, the highest positive cross‐correlation between mood and anxiety across risk groups was observed at Lag 0 (i.e., between a participant's mood and anxiety on the same day), and no statistically significant mood‐anxiety cross‐correlation differences were observed during euthymia.

During depressive episodes, participants who were depressed, had had attempts, and were not actively suicidal showed a stronger negative cross‐correlation (−0.52 (−0.57; −0.36)) than those who were depressed, had no attempts, and were not actively suicidal (−0.37 (−0.44; −0.26); *p* = 0.02). There were no other significant differences.

#### 3.2.3 Cross‐Correlation Between Energy and Anxiety (Lags −7 to 7)

No consistent pattern was observed between energy and anxiety cross‐correlation across risk groups, and the differences across groups were statistically insignificant.

**FIGURE 3:**
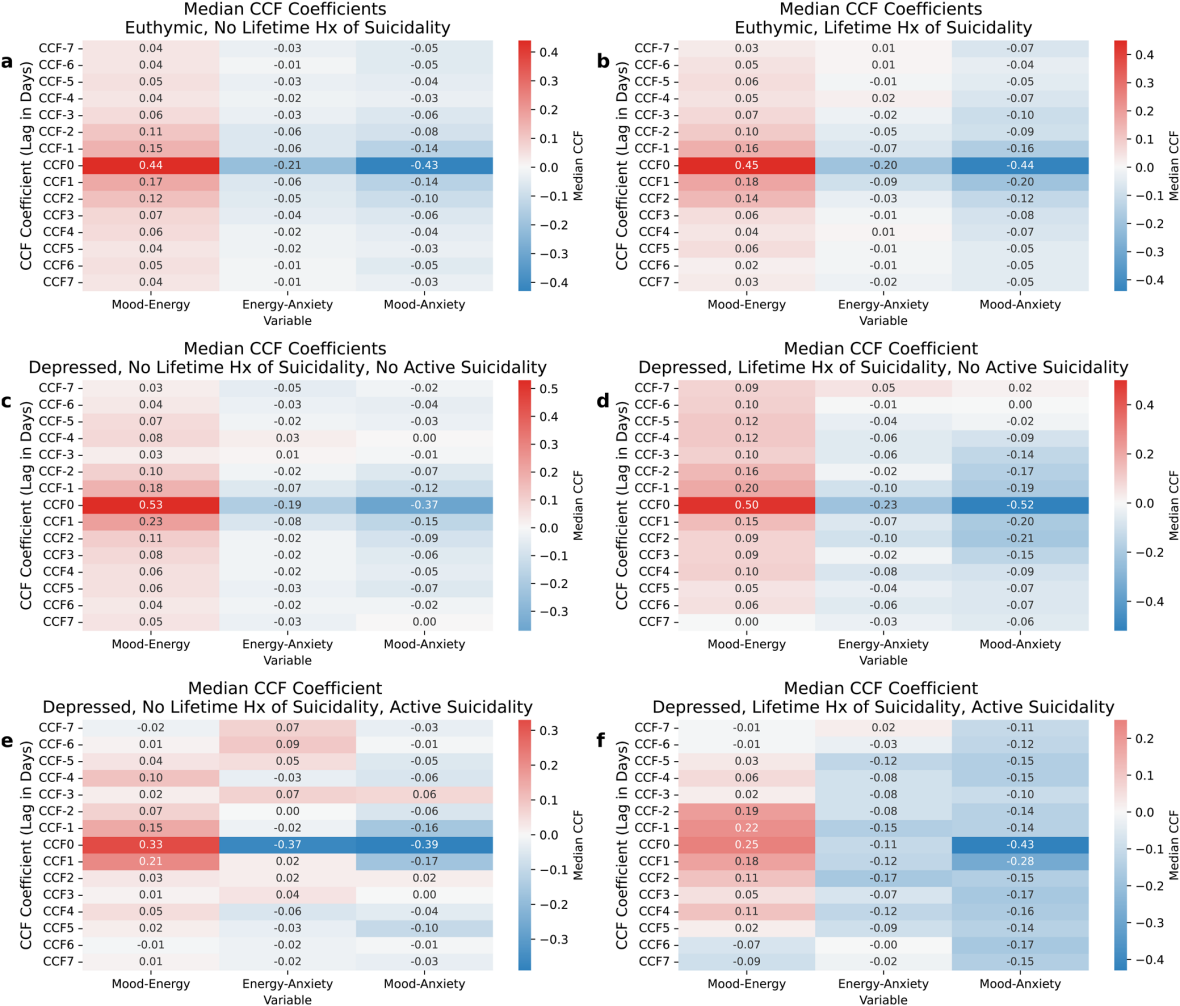
Cross‐correlation analysis matrices (Lags −7 to 7) of mood, energy, and anxiety time series, stratified by increasing risk of suicidality. Cross‐correlation analysis matrices in (a) currently euthymic with no prior suicide attempts; (b) currently euthymic, history of suicide attempts; (c) in a depressive episode, not actively suicidal, no prior suicide attempts; (d) in a depressive episode, not actively suicidal, with a history of suicide attempts; (e) in a depressive episode, actively suicidal, with no prior suicide attempts; and (f) in a depressive episode, actively suicidal, with prior suicidal attempts.

See Figure 3 for the detailed representation of CCF coefficients across classes for the cross‐correlation comparative statistics at all lags (−7 to 7) across polarity and risk groups.

## 4 Discussion

We examined intra‐individual variability in mood regulation by analyzing daily self‐reported mood, anxiety, and energy levels among participants diagnosed with BD, stratified by (i) presence or absence of a prior suicide attempt; (ii) current polarity; and (iii) active suicidality. Our results showed that mood regulation differs across the suicidality continuum. Specifically, participants categorized as high‐risk (i.e., those with both a history of a prior attempt and current suicidal ideation) demonstrated distinctive patterns of mood regulation (i.e., higher autocorrelation and same‐day cross‐correlation for mood, anxiety, and energy series) compared to those classified as low risk (i.e., those without prior suicide attempts and currently euthymic).

While this pattern might reflect the effects of the acute episode (i.e., high anxiety levels coupled with high or low energy levels and low mood), our findings also showed that those participants with prior suicide attempts showed a distinctive, persisting pattern: even when euthymic, this group showed higher day‐to‐day autocorrelation for anxiety. These findings challenge traditional clinical conceptualizations that equate euthymia with the absence of suicide risk and suggest persistent neurobiological vulnerability despite apparent symptomatic remission. Similarly, during depressive episodes, participants in the highest‐risk group (i.e., prior attempts and active suicidal ideation), showed the highest day‐to‐day autocorrelation in anxiety compared to depressed participants with no active suicidal ideation and no prior attempts. Clinically, these findings might promote a more aggressive treatment of comorbid anxiety in those with prior attempts to dampen the risk of future attempts, supporting indirect evidence from RCTs examining the effect of treating anxiety on clinical outcomes in BD [75].

In parallel, the effects of a prior attempt were reflected in higher cross‐correlation between mood and energy in those participants who were currently depressed, even though they were not actively suicidal. Also, in keeping with our finding of a higher day‐to‐day autocorrelation in anxiety in those with prior attempts, even when euthymic, there was a stronger negative cross‐correlation between mood and anxiety in those participants with prior attempts. Our results align with existing literature documenting that a history of suicide attempt may leave physiological sequelae affecting multiple biological systems [76]. Furthermore, they highlight the possible influence of the autonomic nervous system on mood regulation.

Strengths of our study are both methodological and clinical, and include: (i) application of time‐series analysis to suicidal risk continuum in BD; (ii) longitudinal design with densely‐sampled data; (iii) large dataset with 64,351 complete observations across 164 participants; (iv) weekly follow‐up for 6 months in participants recently discharged from an inpatient unit due to a suicide attempt, with low study attrition and high adherence to study protocol. There are several limitations of our study. While our overall sample included 164 participants, the rarity of suicide attempts in real‐world settings [77], inherently creates challenges for achieving balanced group sizes in prospective observational studies. This imbalance reduces statistical power to detect differences, potentially leading to wider confidence intervals for smaller groups. The small sample sizes in the highest‐risk groups may also reduce the generalizability of our findings and increase susceptibility to outlier effects. However, several factors mitigate these concerns: our use of bootstrap resampling methods to estimate robust confidence intervals, the extended observation period, which provided dense temporal data (430 person‐days), and the large effect sizes observed (Cohen's *d* ≥ 1.3) that reached statistical significance despite a small sample size. Nevertheless, replication in larger, more balanced cohorts is essential to confirm these patterns and establish their clinical utility for individual‐level risk prediction. Due to strict inclusion criteria, we were unable to evaluate the impact of certain confounders (e.g., personality disorders, substance abuse). Therefore, our results may not be applicable to a broader clinical population. Finally, we did not collect information on the lethality of the attempt. However, in terms of recency of attempt, participants required admission to a psychiatric unit and were enrolled in the study near the time of their discharge. Our study is clinically relevant as it addresses a critical gap in suicide prediction research; captures dynamic warning signs rather than static risk factors; and challenges the traditional assumption that euthymia equals low suicide risk.

## 5 Conclusion

The burden of suicide in BD makes it imperative to develop better strategies for its prediction and prevention. Our work contributes to the field by capturing the dynamic, state‐dependent nature of mood regulation processes by analyzing densely‐sampled data, and underscoring the role of managing anxiety in the prevention of severe depressive episodes. Our results emphasize that euthymia in BD is not equivalent to the absence of suicidal risk.

Future studies should combine multimodal data (i.e., clinical, physiological) for developing predictive models capable of identifying individual patterns both within individuals (over time) and across individuals. By understanding the dynamic changes in mood, anxiety and energy that characterize different groups at risk for suicide, we will be better positioned to detect the presence of these pervasive patterns and direct personalized interventions. Future research must move beyond correlations toward process‐oriented models that elucidate causal pathways between dysfunction in mood regulatory systems and suicidal vulnerability.

## Author Contributions

Conceptualization: A.O. Methodology: A.O., M.A., and B.H.M. Formal analysis: R.H. Investigation: D.B., C.G.‐T., A.H., M.I.H., M.T., J.Z., M.A., and B.H.M. Resources: A.O. Data curation: M.T. and R.H. Writing – original draft preparation: A.O. Writing – review and editing: A.O., M.A., and B.H.M. Visualization: R.H. and A.H. Funding acquisition: A.O.

## Funding

This work was supported by the Department of Psychiatry Suicide Research Fund at the University of Toronto (AO) and the American Foundation for Suicide Prevention (Grant YIG‐0‐100‐21; Abigail Ortiz).

## Ethics Statement

The authors assert that all procedures contributing to this work comply with the ethical standards of the relevant national and institutional committees on human experimentation and with the Helsinki Declaration of 1975, as revised in 2008.

## Conflicts of Interest

The authors declare no conflicts of interest.

## Supporting information


**Figure S1:** Comparative distributions of the autocorrelation function (ACF) of mood at Lags 1–7 for raw (pre‐imputation) and imputed (post‐imputation) data. Panels (a–f) represent the six suicide risk groups displayed in increasing order of clinical risk.
**Figure S2:** Comparative distributions of the autocorrelation function (ACF) of energy at Lags 1–7 for raw (pre‐imputation) and imputed (post‐imputation) data. Panels (a–f) represent the six suicide risk groups displayed in increasing order of clinical risk.
**Figure S3:** Comparative distributions of the autocorrelation function (ACF) of anxiety at Lags 1–7 for raw (pre‐imputation) and imputed (post‐imputation) data. Panels (a–f) represent the six suicide risk groups displayed in increasing order of clinical risk.
**Figure S4:** Mood autocorrelation association with e‐VAS missing data ratio (Scales 1–7) for Suicide Group 1.
**Figure S5:** Energy autocorrelation association with e‐VAS missing data ratio (Scales 1–7) for Suicide Group 1.
**Figure S6:** Anxiety autocorrelation association with e‐VAS missing data ratio (Scales 1–7) for Suicide Group 1.
**Figure S7:** Mood autocorrelation association with e‐VAS missing data ratio (Scales 1–7) for Suicide Group 2.
**Figure S8:** Energy autocorrelation association with e‐VAS missing data ratio (Scales 1–7) for Suicide Group 2.
**Figure S9:** Anxiety autocorrelation association with e‐VAS missing data ratio (Scales 1–7) for Suicide Group 2.
**Figure S10:** Mood autocorrelation association with e‐VAS missing data ratio (Scales 1–7) for Suicide Group 3.
**Figure S11:** Energy autocorrelation association with e‐VAS missing data ratio (Scales 1–7) for Suicide Group 3.
**Figure S12:** Anxiety autocorrelation association with e‐VAS missing data ratio (Scales 1–7) for Suicide Group 3.
**Figure S13:** Mood, energy, and anxiety missing data ratio longitudinal trends over normalized study course duration (solid blue line: median MDR per segment; shaded blue region: IQR (25%–75%) confidence interval).
**Figure S14:** Missing data ratio autocorrelation (Lags 1–7) across participants (solid blue line: median ACF per lag; shaded blue region: IQR (25%–75%) confidence interval).

## Data Availability

The data that support the findings of this study are available on reasonable request from the corresponding author. The data are not publicly available due to privacy or ethical restrictions.

## References

[1] G. E. Simon , E. Hunkeler , B. Fireman , J. Y. Lee , and J. Savarino , “Risk of Suicide Attempt and Suicide Death in Patients Treated for Bipolar Disorder,” Bipolar Disorders 9, no. 5 (2007): 526–530, 10.1111/j.1399-5618.2007.00408.x. 17680924

[2] Z. Rihmer , X. Gonda , and P. Döme , “The Assessment and Management of Suicide Risk in Bipolar Disorder,” in The Treatment of Bipolar Disorder: Integrative Clinical Strategies and Future Directions (Oxford University Press, 2017), 207–224.

[3] Z. Rihmer , “Suicide Risk in Mood Disorders,” Current Opinion in Psychiatry 20, no. 1 (2007): 17–22, 10.1097/YCO.0b013e3280106868. 17143077

[4] P. Dome , Z. Rihmer , and X. Gonda , “Suicide Risk in Bipolar Disorder: A Brief Review,” Medicina 55, no. 8 (2019): 403, 10.3390/medicina55080403. 31344941 PMC6723289

[5] J. G. Fiedorowicz , J. E. Persons , S. Assari , et al., “Depressive Symptoms Carry an Increased Risk for Suicidal Ideation and Behavior in Bipolar Disorder Without Any Additional Contribution of Mixed Symptoms,” Journal of Affective Disorders 246 (2019): 775–782, 10.1016/j.jad.2018.12.057. 30623823 PMC6914253

[6] A. Schaffer , E. T. Isometsä , L. Tondo , et al., “International Society for Bipolar Disorders Task Force on Suicide: Meta‐Analyses and Meta‐Regression of Correlates of Suicide Attempts and Suicide Deaths in Bipolar Disorder,” Bipolar Disorders 17, no. 1 (2015): 1–16, 10.1111/bdi.12271. PMC6296224 25329791

[7] L. Palagini , G. Cipollone , I. Masci , et al., “Insomnia Symptoms Predict Emotional Dysregulation, Impulsivity and Suicidality in Depressive Bipolar II Patients With Mixed Features,” Comprehensive Psychiatry 89 (2019): 46–51, 10.1016/j.comppsych.2018.12.009. 30593973

[8] I. Sverdlichenko , K. Jansen , L. D. M. Souza , R. A. da Silva , F. Kapczinski , and T. A. Cardoso , “Mixed Episodes and Suicide Risk: A Community Sample of Young Adults,” Journal of Affective Disorders 266 (2020): 252–257, 10.1016/j.jad.2020.01.111. 32056885

[9] T. R. Goldstein , J. Merranko , D. Hafeman , et al., “A Risk Calculator to Predict Suicide Attempts Among Individuals With Early‐Onset Bipolar Disorder,” Focus 21, no. 4 (2023): 412–419, 10.1176/appi.focus.23021023. 38695011 PMC11058951

[10] A. Miola , L. Tondo , M. Pinna , M. Contu , and R. J. Baldessarini , “Suicidal Risk and Protective Factors in Major Affective Disorders: A Prospective Cohort Study of 4307 Participants,” Journal of Affective Disorders 338 (2023): 189–198, 10.1016/j.jad.2023.06.018. 37301296

[11] F. Trémeau , L. Staner , F. Duval , et al., “Suicide Attempts and Family History of Suicide in Three Psychiatric Populations,” Suicide & Life‐Threatening Behavior 35, no. 6 (2005): 702–713, 10.1521/suli.2005.35.6.702. 16552986

[12] A. Pemau , C. Marin‐Martin , M. Diaz‐Marsa , et al., “Risk Factors for Suicide Reattempt: A Systematic Review and Meta‐Analysis,” Psychological Medicine 54, no. 9 (2024): 1897–1904, 10.1017/s0033291724000904. 38623694

[13] K. Yates , U. Lång , M. Cederlöf , et al., “Association of Psychotic Experiences With Subsequent Risk of Suicidal Ideation, Suicide Attempts, and Suicide Deaths: A Systematic Review and Meta‐Analysis of Longitudinal Population Studies,” JAMA Psychiatry 76, no. 2 (2019): 180–189, 10.1001/jamapsychiatry.2018.3514. 30484818 PMC6439738

[14] C. L. Bagge , K. P. Himes , S. M. Cohen , E. V. Barbour , K. A. Comtois , and A. K. Littlefield , “Can Profiles of Behaviors Occurring Within 48 h of a Suicide Attempt Predict Future Severity of Suicidal Thoughts and Reattempt?: An Examination of Hospitalized Patients 12 Months Post‐Discharge,” Journal of Psychiatric Research 176 (2024): 259–264, 10.1016/j.jpsychires.2024.06.022. 38901390

[15] A. A. M. Hubers , S. Moaddine , S. H. M. Peersmann , et al., “Suicidal Ideation and Subsequent Completed Suicide in Both Psychiatric and Non‐Psychiatric Populations: A Meta‐Analysis,” Epidemiology and Psychiatric Sciences 27, no. 2 (2018): 186–198, 10.1017/s2045796016001049. 27989254 PMC6998965

[16] R. K. Morriss , M. A. Faizal , A. P. Jones , P. R. Williamson , C. Bolton , and J. P. McCarthy , “Interventions for Helping People Recognise Early Signs of Recurrence in Bipolar Disorder,” Cochrane Database of Systematic Reviews 2007, no. 1 (2007): Cd004854, 10.1002/14651858.CD004854.pub2. 17253526 PMC6544804

[17] L. Tondo , G. H. Vázquez , and R. J. Baldessarini , “Prevention of Suicidal Behavior in Bipolar Disorder,” Bipolar Disorders 23, no. 1 (2021): 14–23, 10.1111/bdi.13017. 33037692

[18] J. C. Franklin , J. D. Ribeiro , K. R. Fox , et al., “Risk Factors for Suicidal Thoughts and Behaviors: A Meta‐Analysis of 50 Years of Research,” Psychological Bulletin 143, no. 2 (2017): 187–232, 10.1037/bul0000084. 27841450

[19] L. Kivelä , W. A. J. van der Does , H. Riese , and N. Antypa , “Don't Miss the Moment: A Systematic Review of Ecological Momentary Assessment in Suicide Research. Systematic Review,” Frontiers in Digital Health 4 (2022): 876595, 10.3389/fdgth.2022.876595. 35601888 PMC9120419

[20] C. A. Claassen , J. D. Harvilchuck‐Laurenson , and J. Fawcett , “Prognostic Models to Detect and Monitor the Near‐Term Risk of Suicide: State of the Science,” American Journal of Preventive Medicine 47, no. 3 Suppl 2 (2014): S181–S185, 10.1016/j.amepre.2014.06.003. 25145737

[21] M. Pompili , X. Gonda , G. Serafini , et al., “Epidemiology of Suicide in Bipolar Disorders: A Systematic Review of the Literature,” Bipolar Disorders 15, no. 5 (2013): 457–490, 10.1111/bdi.12087. 23755739

[22] S. B. Wang , D. D. L. Coppersmith , E. M. Kleiman , et al., “A Pilot Study Using Frequent Inpatient Assessments of Suicidal Thinking to Predict Short‐Term Postdischarge Suicidal Behavior,” JAMA Network Open 4, no. 3 (2021): e210591, 10.1001/jamanetworkopen.2021.0591. 33687442 PMC7944382

[23] E. M. Kleiman , B. J. Turner , S. Fedor , E. E. Beale , J. C. Huffman , and M. K. Nock , “Examination of Real‐Time Fluctuations in Suicidal Ideation and Its Risk Factors: Results From Two Ecological Momentary Assessment Studies,” Journal of Abnormal Psychology 126, no. 6 (2017): 726–738, 10.1037/abn0000273. 28481571

[24] E. K. Czyz , H. J. Koo , N. Al‐Dajani , S. D. Kentopp , A. Jiang , and C. A. King , “Temporal Profiles of Suicidal Thoughts in Daily Life: Results From Two Mobile‐Based Monitoring Studies With High‐Risk Adolescents,” Journal of Psychiatric Research 153 (2022): 56–63, 10.1016/j.jpsychires.2022.06.050. 35797815 PMC9811520

[25] M. Kamali , N. A. Reilly‐Harrington , W. C. Chang , et al., “Bipolar Depression and Suicidal Ideation: Moderators and Mediators of a Complex Relationship,” Journal of Affective Disorders 259 (2019): 164–172, 10.1016/j.jad.2019.08.032. 31445343

[26] M. Kamali , E. F. H. Saunders , S. Assari , K. A. Ryan , D. F. Marshall , and M. G. McInnis , “Mood, Dimensional Personality, and Suicidality in a Longitudinal Sample of Patients With Bipolar Disorder and Controls,” Suicide and Life‐Threatening Behavior 49, no. 5 (2019): 1360–1378, 10.1111/sltb.12529. 30450613

[27] L. Palagini , G. Cipollone , U. Moretto , et al., “Chronobiological Dis‐Rhythmicity Is Related to Emotion Dysregulation and Suicidality in Depressive Bipolar II Disorder With Mixed Features,” Psychiatry Research 271 (2019): 272–278, 10.1016/j.psychres.2018.11.056. 30508671

[28] M. K. Titone , N. Goel , T. H. Ng , L. E. MacMullen , and L. B. Alloy , “Impulsivity and Sleep and Circadian Rhythm Disturbance Predict Next‐Day Mood Symptoms in a Sample at High Risk for or With Recent‐Onset Bipolar Spectrum Disorder: An Ecological Momentary Assessment Study,” Journal of Affective Disorders 298 (2022): 17–25, 10.1016/j.jad.2021.08.155. 34728283 PMC8643329

[29] M. H. Huang , Y. H. Kuan , P. C. Tu , W. C. Chang , Y. E. Chan , and T. P. Su , “Altered Functional Connectivity of Prefrontal Cortex‐Related Circuitry and Trait Impulsivity in Patients With Bipolar Disorder and History of Suicide Attempts,” Acta Psychiatrica Scandinavica 151, no. 5 (2025): 634–643, 10.1111/acps.13786. 39756804

[30] M. F. Armey , L. Brick , H. T. Schatten , N. R. Nugent , and I. W. Miller , “Ecologically Assessed Affect and Suicidal Ideation Following Psychiatric Inpatient Hospitalization,” General Hospital Psychiatry 63 (2020): 89–96, 10.1016/j.genhosppsych.2018.09.008. 30297091 PMC6581626

[31] N. Hallensleben , H. Glaesmer , T. Forkmann , et al., “Predicting Suicidal Ideation by Interpersonal Variables, Hopelessness and Depression in Real‐Time. An Ecological Momentary Assessment Study in Psychiatric Inpatients With Depression,” European Psychiatry 56 (2019): 43–50, 10.1016/j.eurpsy.2018.11.003. 30530103

[32] I. H. Stanley , J. W. Boffa , M. L. Rogers , et al., “Anxiety Sensitivity and Suicidal Ideation/Suicide Risk: A Meta‐Analysis,” Journal of Consulting and Clinical Psychology 86, no. 11 (2018): 946–960, 10.1037/ccp0000342. 30335426 PMC6469498

[33] A. Kanwar , S. Malik , L. J. Prokop , et al., “The Association Between Anxiety Disorders and Suicidal Behaviors: A Systematic Review and Meta‐Analysis,” Depression and Anxiety 30, no. 10 (2013): 917–929, 10.1002/da.22074. 23408488

[34] K. K. Patel , J. C. Kearns , D. Foti , W. R. Pigeon , E. M. Kleiman , and C. R. Glenn , “Anhedonia Links Sleep Problems and Suicidal Thoughts: An Intensive Longitudinal Study in High‐Risk Adolescents,” Research on Child and Adolescent Psychopathology 53, no. 3 (2025): 331–347, 10.1007/s10802-024-01275-w. 39680285 PMC11913912

[35] R. C. Cox , S. L. Brown , B. N. Chalmers , and L. N. Scott , “Examining Sleep Disturbance Components as Near‐Term Predictors of Suicide Ideation in Daily Life,” Psychiatry Research 326 (2023): 115323, 10.1016/j.psychres.2023.115323. 37392522 PMC10527974

[36] G. I. Teresi , J. Merranko , G. Porta , et al., “Worsening Sleep Predicts Next‐Week Suicidal Ideation in a High‐Risk Adolescent Outpatient Treatment Sample,” Suicide and Life‐Threatening Behavior 55, no. 2 (2025): e13141, 10.1111/sltb.13141. 39498740 PMC11879923

[37] I. Berardelli , S. Sarubbi , M. A. Trocchia , et al., “The Mediating Role of Insomnia Severity in the Relationship Between Anxiety Symptoms and Suicidal Ideation,” Journal of Nervous and Mental Disease 212, no. 9 (2024): 479–484, 10.1097/nmd.0000000000001793. 39120957

[38] C. Chu , J. A. Nota , A. L. Silverman , C. Beard , and T. Björgvinsson , “Pathways Among Sleep Onset Latency, Relationship Functioning, and Negative Affect Differentiate Patients With Suicide Attempt History From Patients With Suicidal Ideation,” Psychiatry Research 273 (2019): 788–797, 10.1016/j.psychres.2018.11.014. 31207867

[39] I. Vargas , M. L. Perlis , M. Grandner , et al., “Insomnia Symptoms and Suicide‐Related Ideation in US Army Service Members,” Behavioral Sleep Medicine 18, no. 6 (2020): 820–836, 10.1080/15402002.2019.1693373. 31738588

[40] A. S. Holdaway , A. M. Luebbe , and S. P. Becker , “Rumination in Relation to Suicide Risk, Ideation, and Attempts: Exacerbation by Poor Sleep Quality?,” Journal of Affective Disorders 236 (2018): 6–13, 10.1016/j.jad.2018.04.087. 29704657 PMC6047760

[41] C. A. Depp , W. K. Thompson , E. Frank , and H. A. Swartz , “Prediction of Near‐Term Increases in Suicidal Ideation in Recently Depressed Patients With Bipolar II Disorder Using Intensive Longitudinal Data,” Journal of Affective Disorders 208 (2017): 363–368. 27810719 10.1016/j.jad.2016.09.054 PMC5154812

[42] W. K. Thompson , A. Gershon , R. O'Hara , R. A. Bernert , and C. A. Depp , “The Prediction of Study‐Emergent Suicidal Ideation in Bipolar Disorder: A Pilot Study Using Ecological Momentary Assessment Data,” Bipolar Disorders 16, no. 7 (2014): 669–677, 10.1111/bdi.12218. 24903771 PMC4213287

[43] M. Sanches , L. K. Nguyen , T. H. Chung , et al., “Anxiety Symptoms and Suicidal Thoughts and Behaviors Among Patients With Mood Disorders,” Journal of Affective Disorders 307 (2022): 171–177, 10.1016/j.jad.2022.03.046. 35331824 PMC9321173

[44] A. Ortiz and M. Alda , “The Perils of Being Too Stable: Mood Regulation in Bipolar Disorder,” Journal of Psychiatry & Neuroscience 43, no. 6 (2018): 363–365, 10.1503/jpn.180183. 30371048 PMC6203548

[45] C. L. Ehlers , “Chaos and Complexity. Can It Help Us to Understand Mood and Behavior?,” Archives of General Psychiatry 52, no. 11 (1995): 960–964. 7487344 10.1001/archpsyc.1995.03950230074010

[46] S. M. Pincus , “Assessing Serial Irregularity and Its Implications for Health,” Annals of the New York Academy of Sciences 954 (2001): 245–267. 11797860 10.1111/j.1749-6632.2001.tb02755.x

[47] C. Chatfield , The Analysis of Time Series: An Introduction (CRC press, 2016).

[48] V. K. Yeragani and V. Sree Hari Rao , “Patterns of Oscillatory Behavior in Different Human Systems: A Special Reference to Psychiatry and Techniques to Quantify Such Patterns,” Bipolar Disorders 8, no. 5 (2006): 421–422, 10.1111/j.1399-5618.2006.00377.x. 17042878

[49] V. K. Yeragani , R. Pohl , M. Mallavarapu , and R. Balon , “Approximate Entropy of Symptoms of Mood: An Effective Technique to Quantify Regularity of Mood,” Bipolar Disorders 5, no. 4 (2003): 279–286. 12895205 10.1034/j.1399-5618.2003.00012.x

[50] H. F. Jelinek , H. Md Imam , H. Al‐Aubaidy , and A. H. Khandoker , “Association of Cardiovascular Risk Using Non‐Linear Heart Rate Variability Measures With the Framingham Risk Score in a Rural Population,” Frontiers in Physiology 4 (2013): 186, 10.3389/fphys.2013.00186. 23898302 PMC3724049

[51] H. T. Wu , C. C. Liu , M. T. Lo , et al., “Multiscale Cross‐Approximate Entropy Analysis as a Measure of Complexity Among the Aged and Diabetic,” Computational and Mathematical Methods in Medicine 2013 (2013): 324325, 10.1155/2013/324325. 23864905 PMC3705813

[52] R. W. Cowdry , D. L. Gardner , K. M. O'Leary , E. Leibenluft , and D. R. Rubinow , “Mood Variability: A Study of Four Groups,” Americal Journal of Psychiatry 148, no. 11 (1991): 1505–1511, 10.1176/ajp.148.11.1505. 1928464

[53] J. A. Golier , R. Yehuda , J. Schmeidler , and L. J. Siever , “Variability and Severity of Depression and Anxiety in Post Traumatic Stress Disorder and Major Depressive Disorder,” Depression and Anxiety 13, no. 2 (2001): 97–100. 11301926 10.1002/da.1022

[54] D. Katerndahl , R. Ferrer , R. Best , and C. P. Wang , “Dynamic Patterns in Mood Among Newly Diagnosed Patients With Major Depressive Episode or Panic Disorder and Normal Controls,” Primary Care Companion to the Journal of Clinical Psychiatry 9, no. 3 (2007): 183–187. 10.4088/pcc.v09n0303 PMC1911176 17632650

[55] S. M. Pincus , P. J. Schmidt , P. Palladino‐Negro , and D. R. Rubinow , “Differentiation of Women With Premenstrual Dysphoric Disorder, Recurrent Brief Depression, and Healthy Controls by Daily Mood Rating Dynamics,” Journal of Psychiatric Research 42, no. 5 (2008): 337–347, 10.1016/j.jpsychires.2007.01.001. 17336329

[56] A. Gottschalk , M. S. Bauer , and P. C. Whybrow , “Evidence of Chaotic Mood Variation in Bipolar Disorder,” Archives of General Psychiatry 52, no. 11 (1995): 947–959. 7487343 10.1001/archpsyc.1995.03950230061009

[57] A. Ortiz , K. Bradler , J. Garnham , C. Slaney , and M. Alda , “Nonlinear Dynamics of Mood Regulation in Bipolar Disorder,” Bipolar Disorders 17, no. 2 (2015): 139–149. 25118155 10.1111/bdi.12246

[58] A. Ortiz , K. Bradler , J. Garnham , C. Slaney , S. MacLean , and M. Alda , “Corrigendum to Nonlinear Dynamics of Mood Regulation in Unaffected First‐Degree Relatives of Bipolar Disorder Patients,” Journal of Affective Disorders 245 (2019): 16. 30366232 10.1016/j.jad.2018.10.103

[59] R. Halabi , K. Yusuff , C. Park , et al., “Mood Regulation in Euthymic Patients With a History of Antidepressant‐Induced Mania,” Bipolar Disorders 26 (2024): 810–819. 39333012 10.1111/bdi.13504 PMC11627008

[60] A. Ortiz , A. Hintze , R. Burnett , et al., “Identifying Patient‐Specific Behaviors to Understand Illness Trajectories and Predict Relapses in Bipolar Disorder Using Passive Sensing and Deep Anomaly Detection: Protocol for a Contactless Cohort Study,” BMC Psychiatry 22, no. 1 (2022): 288, 10.1186/s12888-022-03923-1. 35459150 PMC9026652

[61] Diagnostic and Statistical Manual of Mental Disorders, 5th ed. (American Psychiatric Association, 2013).

[62] M. First , J. Williams , R. Karg , and R. L. Spitzer , Structured Clinical Interview for DSM‐5, Research Version (SCID‐5) (American Psychiatric Association, 2015).

[63] R. C. Young , J. T. Biggs , V. E. Ziegler , and D. A. Meyer , “A Rating Scale for Mania: Reliability, Validity and Sensitivity,” British Journal of Psychiatry 133 (1978): 429–435. 10.1192/bjp.133.5.429 728692

[64] S. A. Montgomery and M. Asberg , “A New Depression Scale Designed to Be Sensitive to Change,” British Journal of Psychiatry 134 (1979): 382–389. 10.1192/bjp.134.4.382 444788

[65] E. Frank , R. F. Prien , R. B. Jarrett , et al., “Conceptualization and Rationale for Consensus Definitions of Terms in Major Depressive Disorder. Remission, Recovery, Relapse, and Recurrence,” Archives of General Psychiatry 48, no. 9 (1991): 851–855, 10.1001/archpsyc.1991.01810330075011. 1929776

[66] C. J. Hawley , T. M. Gale , and T. Sivakumaran , “Defining Remission by Cut Off Score on the MADRS: Selecting the Optimal Value,” Journal of Affective Disorders 72, no. 2 (2002): 177–184, 10.1016/S0165-0327(01)00451-7. 12200208

[67] M. Zimmerman , M. A. Posternak , and I. Chelminski , “Defining Remission on the Montgomery‐Asberg Depression Rating Scale,” Journal of Clinical Psychiatry 65, no. 2 (2004): 163–168, 10.4088/jcp.v65n0204. 15003068

[68] M. Tohen , J. R. Calabrese , G. S. Sachs , et al., “Randomized, Placebo‐Controlled Trial of Olanzapine as Maintenance Therapy in Patients With Bipolar I Disorder Responding to Acute Treatment With Olanzapine,” American Journal of Psychiatry 163, no. 2 (2006): 247–256, 10.1176/appi.ajp.163.2.247. 16449478

[69] E. Vieta , T. Suppes , I. Eggens , I. Persson , B. Paulsson , and M. Brecher , “Efficacy and Safety of Quetiapine in Combination With Lithium or Divalproex for Maintenance of Patients With Bipolar I Disorder (International Trial 126),” Journal of Affective Disorders 109, no. 3 (2008): 251–263, 10.1016/j.jad.2008.06.001. 18579216

[70] P. A. Harris , R. Taylor , R. Thielke , J. Payne , N. Gonzalez , and J. G. Conde , “Research Electronic Data Capture (REDCap)—A Metadata‐Driven Methodology and Workflow Process for Providing Translational Research Informatics Support,” Journal of Biomedical Informatics 42, no. 2 (2009): 377–381, 10.1016/j.jbi.2008.08.010. 18929686 PMC2700030

[71] D. A. Dickey and W. A. Fuller , “Distribution of the Estimators for Autoregressive Time Series With a Unit Root,” Journal of the American Statistical Association 74, no. 366 (1979): 427–431, 10.2307/2286348.

[72] R. Halabi , B. H. Mulsant , M. Alda , et al., “Not Missing at Random: Missing Data Are Associated With Clinical Status and Trajectories in an Electronic Monitoring Longitudinal Study of Bipolar Disorder,” Journal of Psychiatric Research 174 (2024): 326–331, 10.1016/j.jpsychires.2024.04.036. 38692162 PMC11295604

[73] S. Zhang , “Nearest Neighbor Selection for Iteratively kNN Imputation,” Journal of Systems and Software 85, no. 11 (2012): 2541–2552, 10.1016/j.jss.2012.05.073.

[74] R. A. Fisher , “Frequency Distribution of the Values of the Correlation Coefficient in Samples From an Indefinitely Large Population,” Biometrika 10, no. 4 (1915): 507–521, 10.2307/2331838.

[75] L. N. Yatham , S. H. Kennedy , S. V. Parikh , et al., “Canadian Network for Mood and Anxiety Treatments (CANMAT) and International Society for Bipolar Disorders (ISBD) 2018 Guidelines for the Management of Patients With Bipolar Disorder,” Bipolar Disorders 20, no. 2 (2018): 97–170, 10.1111/bdi.12609. 29536616 PMC5947163

[76] A. Ortiz , Y. Park , S. MacLean , et al., “A History of Suicide Attempt Is Associated With Increased Sympathetic Activation in Bipolar Disorder,” Canadian Journal of Psychiatry 69, no. 2 (2024): 126–137, 10.1177/07067437231194334. 37583363 PMC10789230

[77] C. Kitchen , A. Zirikly , A. Belouali , H. Kharrazi , P. Nestadt , and H. C. Wilcox , “Suicide Death Prediction Using the Maryland Suicide Data Warehouse: A Sensitivity Analysis,” Archives of Suicide Research 29, no. 2 (2025): 453–467, 10.1080/13811118.2024.2363227. 38945167 PMC12001816

