# Supplementary material for: Trajectories of Suicidal Risk Impact Mood Regulation Differently in Patients With a Diagnosis of Bipolar Disorder
Source: Acta Psychiatr Scand. 2026 Apr 2;154(1):60–73. doi: 10.1111/acps.70094 (PMC13242970; doi:10.1111/acps.70094)
Supplement: Supplementary file 1 — Figure S1: Comparative distributions of the autocorrelation function (ACF) of mood at Lags 1–7 for raw (pre‐imputation) and imputed (post‐imputation) data. Panels (a–f) represent the six suicide risk groups displayed in increasing order of clinical risk. Figure S2: Comparative distributions of the autocorrelation function (ACF) of energy at Lags 1–7 for raw (pre‐imputation) and imputed (post‐imputation) data. Panels (a–f) represent the six suicide risk groups displayed in increasing order of clinical risk. Figure S3: Comparative distributions of the autocorrelation function (ACF) of anxiety at Lags 1–7 for raw (pre‐imputation) and imputed (post‐imputation) data. Panels (a–f) represent the six suicide risk groups displayed in increasing order of clinical risk. Figure S4: Mood autocorrelation association with e‐VAS missing data ratio (Scales 1–7) for Suicide Group 1. Figure S5: Energy autocorrelation association with e‐VAS missing data ratio (Scales 1–7) for Suicide Group 1. Figure S6: Anxiety autocorrelation association with e‐VAS missing data ratio (Scales 1–7) for Suicide Group 1. Figure S7: Mood autocorrelation association with e‐VAS missing data ratio (Scales 1–7) for Suicide Group 2. Figure S8: Energy autocorrelation association with e‐VAS missing data ratio (Scales 1–7) for Suicide Group 2. Figure S9: Anxiety autocorrelation association with e‐VAS missing data ratio (Scales 1–7) for Suicide Group 2. Figure S10: Mood autocorrelation association with e‐VAS missing data ratio (Scales 1–7) for Suicide Group 3. Figure S11: Energy autocorrelation association with e‐VAS missing data ratio (Scales 1–7) for Suicide Group 3. Figure S12: Anxiety autocorrelation association with e‐VAS missing data ratio (Scales 1–7) for Suicide Group 3. Figure S13: Mood, energy, and anxiety missing data ratio longitudinal trends over normalized study course duration (solid blue line: median MDR per segment; shaded blue region: IQR (25%–75%) confidence interval). Figure S14: Missing data ra [file ACPS-154-60-s001.docx]

**Supplementary Methods**

We implemented a series of supplementary analyses to assess the robustness of our time series analyses against data completeness, missing data imputation technique, and suicide risk stratification.

Data completeness sensitivity analysis:

To assess the impact of data completion on temporal dynamics, we calculated the Autocorrelation Function (ACF) for both the raw and imputed time series. Specifically, we conducted a sensitivity analysis by comparing our primary results against alternative imputation models (Suppl. Figures 1-3). To evaluate sensitivity to the imputation strategy, we compared our time series analyses’ results with no imputation (complete-case/observed-only data) against Multiple Imputation by Chained Equations (MICE), then compared the resulting distributions of the ACF under each approach (e.g., density/summary statistics across participants and risk groups) and assessed distributional differences.

Data completeness association with suicide risk group:

To assess the potential for systematic bias in data completeness, we investigated the associations between Missing Data Ratios (MDR) and the temporal dynamics of mood, energy, and anxiety (Suppl. Table 1). We employed a robust statistical framework utilizing Kruskal-Wallis tests to compare MDR distributions across six distinct suicidality cohorts, supplemented by pairwise Mann-Whitney U tests with Holm-Bonferroni corrections to identify group-specific differences. Furthermore, we evaluated the relationship between data missingness and signal properties by calculating the correlation between MDR and both ACF and CCF coefficients across all e-VAS variables and temporal lags (Suppl. Figures 4-12).

Longitudinal Missing Data Ratio dynamics:

To characterize the temporal structure of missing data across participants with heterogeneous follow-up durations, we implemented a sliding-window approach on a normalized time axis. For each participant, we first converted the time series into a daily binary missingness indicator (1 = missing, 0 = observed) by checking whether any valid observation existed on each calendar day within the participant's monitoring period. We then applied a rolling window to compute the MDR at each time point, where the window size was set as a fixed percentage of each participant's total duration (default 10%, clamped between 7 and 30 days to balance temporal resolution and stability). To enable aggregation across participants with different study course lengths, we mapped each participant's timeline onto a normalized 0–100% scale representing their proportional progress through follow-up, with the rolling MDR values assigned to percent-bins based on the window center. Finally, we aggregated MDR trajectories across all participants by computing the median and interquartile range at each percent-bin, producing a group-level longitudinal MDR profile that reveals whether missingness is stable, increases/decreases over time, or exhibits temporal clustering independent of absolute date and time (Suppl. Figures 13-14).

**Supplementary Results**

Data completeness sensitivity analysis:


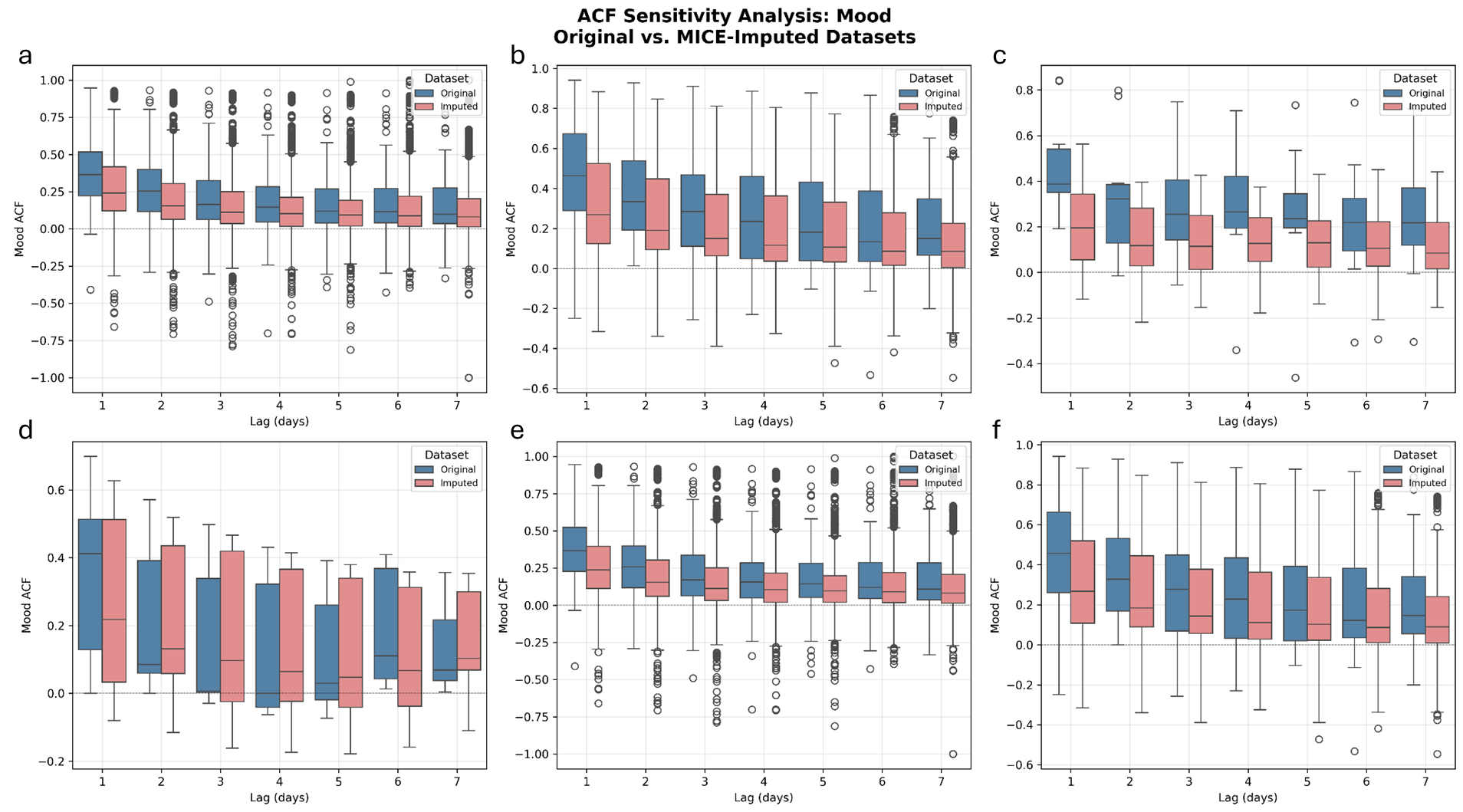


**Supplementary Figure 1.** Comparative distributions of the Autocorrelation Function (ACF) of Mood at lags 1 to 7 for raw (pre-imputation) and imputed (post-imputation) data. Panels (a–f) represent the six suicide risk groups displayed in increasing order of clinical risk.


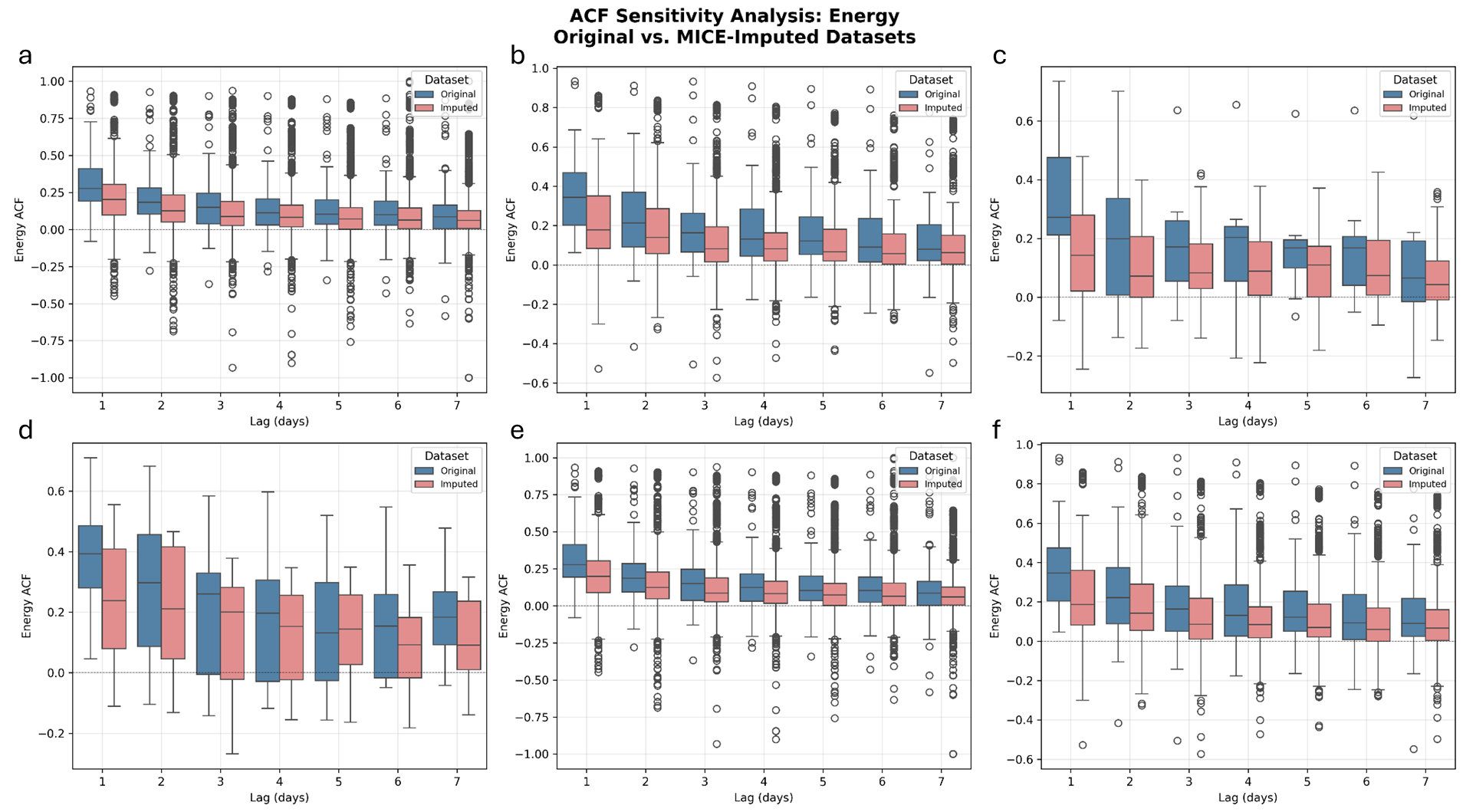


**Supplementary Figure 2.** Comparative distributions of the Autocorrelation Function (ACF) of Energy at lags 1 to 7 for raw (pre-imputation) and imputed (post-imputation) data. Panels (a–f) represent the six suicide risk groups displayed in increasing order of clinical risk.


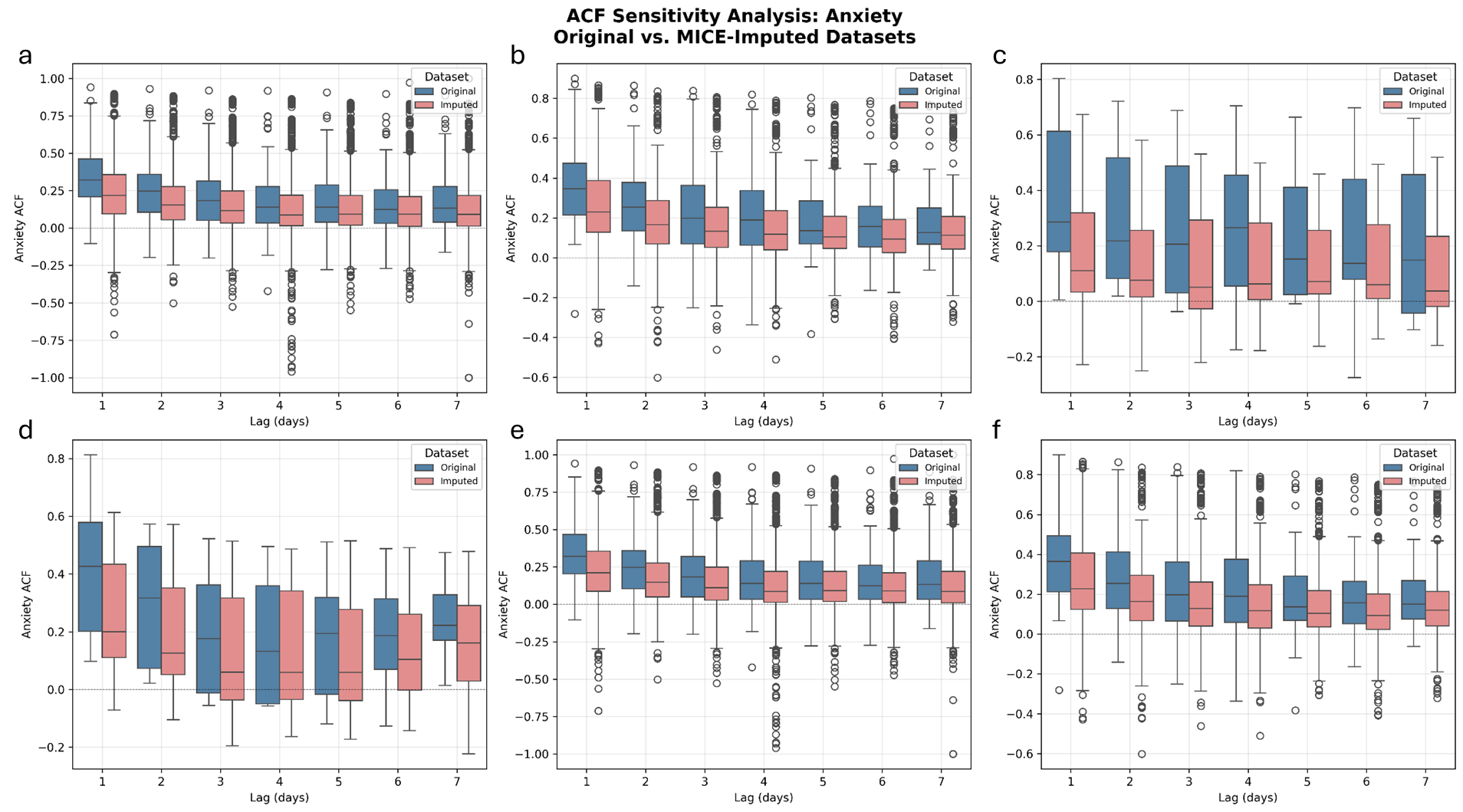


**Supplementary Figure 3.** Comparative distributions of the Autocorrelation Function (ACF) of Anxiety at lags 1 to 7 for raw (pre-imputation) and imputed (post-imputation) data. Panels (a–f) represent the six suicide risk groups displayed in increasing order of clinical risk.

Data completeness association with suicide risk group:

The Kruskal–Wallis tests revealed no statistically significant differences in missing data ratios (MDR) across the suicidality groups for daily self-reported e-VAS scales (H < 0.01, p > .05). Post hoc pairwise comparisons using Mann–Whitney U tests further confirmed that data completeness remained consistent across all groups, with no significant differences observed after Holm–Bonferroni correction (all adjusted p=1.00) and negligible effect sizes (all Cliff’s ∣δ∣<0.08|\delta| < 0.08∣δ∣<0.08). These results suggest that the data completeness was not biased by the participants' suicide risk level.

Supplementary Table 1: Pairwise Comparisons of Missing Data Ratios (MDR) Across Suicide Risk Groups

| Group A | Group B | Mann-Whitney U | p-value (Holm-corrected) | Cliff's Delta |
| --- | --- | --- | --- | --- |
| 1 | 2 | 2623.5 | > 0.05 | -0.003 |
| 1 | 3 | 6844.5 | > 0.05 | 0.000 |
| 1 | 4 | 2623.5 | > 0.05 | -0.003 |
| 1 | 5 | 5343.5 | > 0.05 | 0.001 |
| 1 | 6 | 3523.5 | > 0.05 | 0.003 |
| 2 | 3 | 2641.5 | > 0.05 | 0.003 |
| 2 | 4 | 1012.5 | > 0.05 | 0.000 |
| 2 | 5 | 5242.5 | > 0.05 | 0.001 |
| 2 | 6 | 3424.5 | > 0.05 | 0.003 |
| 3 | 4 | 2623.5 | > 0.05 | -0.003 |
| 3 | 5 | 3253.5 | > 0.05 | 0.002 |
| 3 | 6 | 2424.5 | > 0.05 | 0.003 |

**Statistical significance was assessed using Mann-Whitney U tests with Holm-Bonferroni correction for multiple comparisons. Cliff's Delta was used to estimate effect size (0.147 = small, 0.33 = medium, 0.474 = large). No significant differences in missingness were found between any pair of groups.*


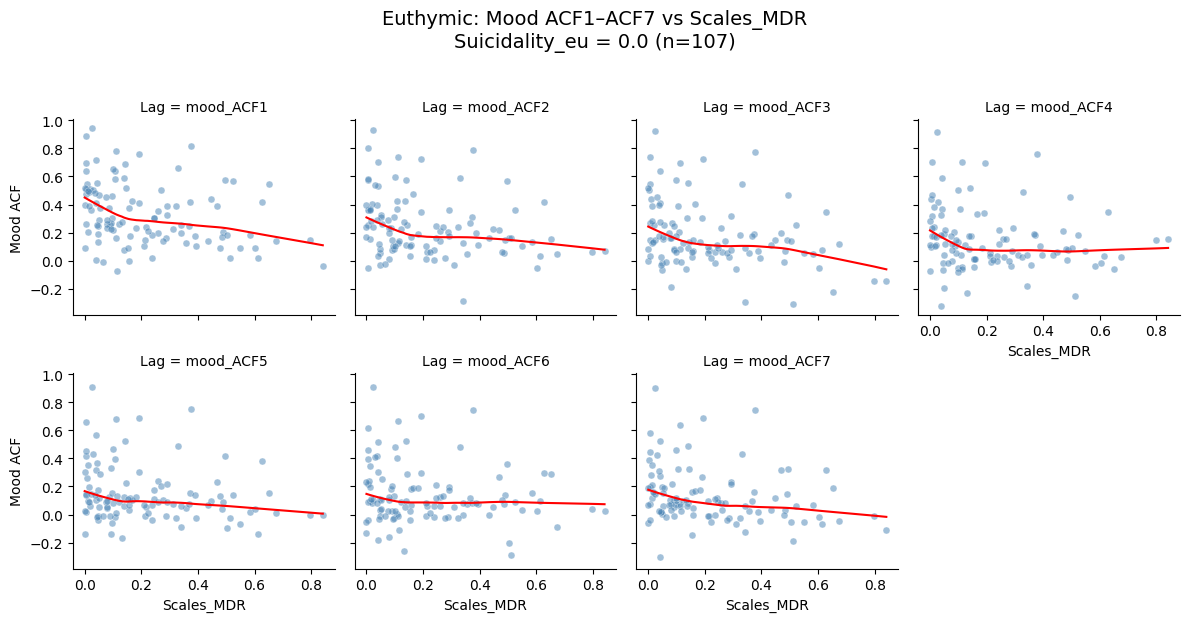


**Supplementary Figure 4.** Mood autocorrelation association with e-VAS missing data ratio (scales 1 to 7) for Suicide Group 1.


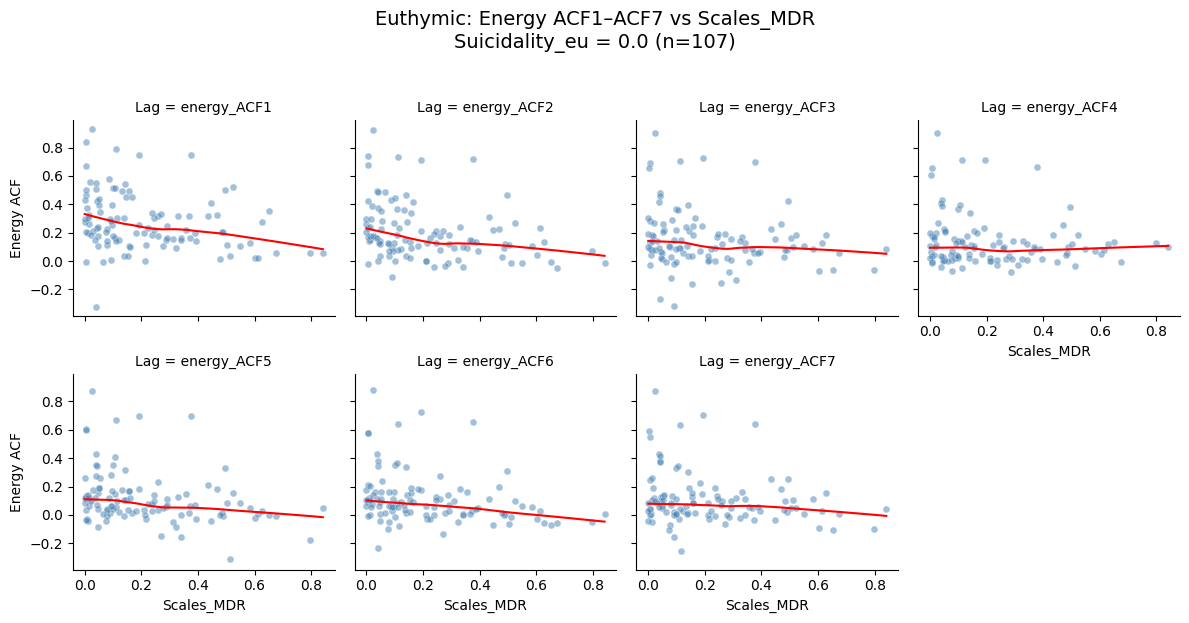


**Supplementary Figure 5.** Energy autocorrelation association with e-VAS missing data ratio (scales 1 to 7) for Suicide Group 1.


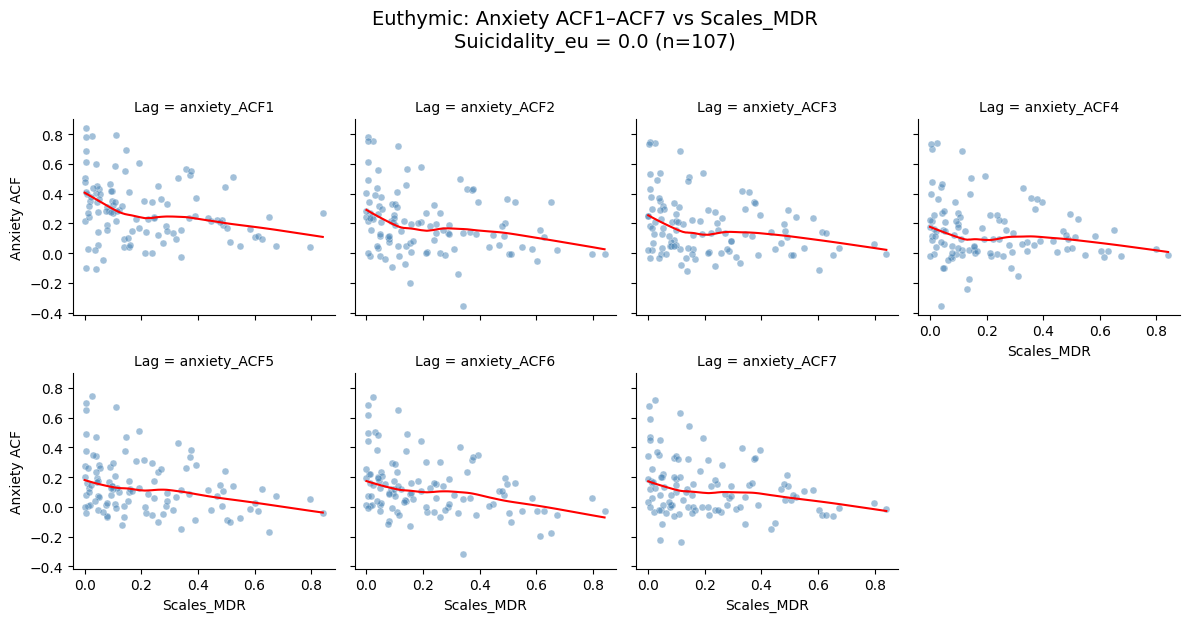


**Supplementary Figure 6.** Anxiety autocorrelation association with e-VAS missing data ratio (scales 1 to 7) for Suicide Group 1.


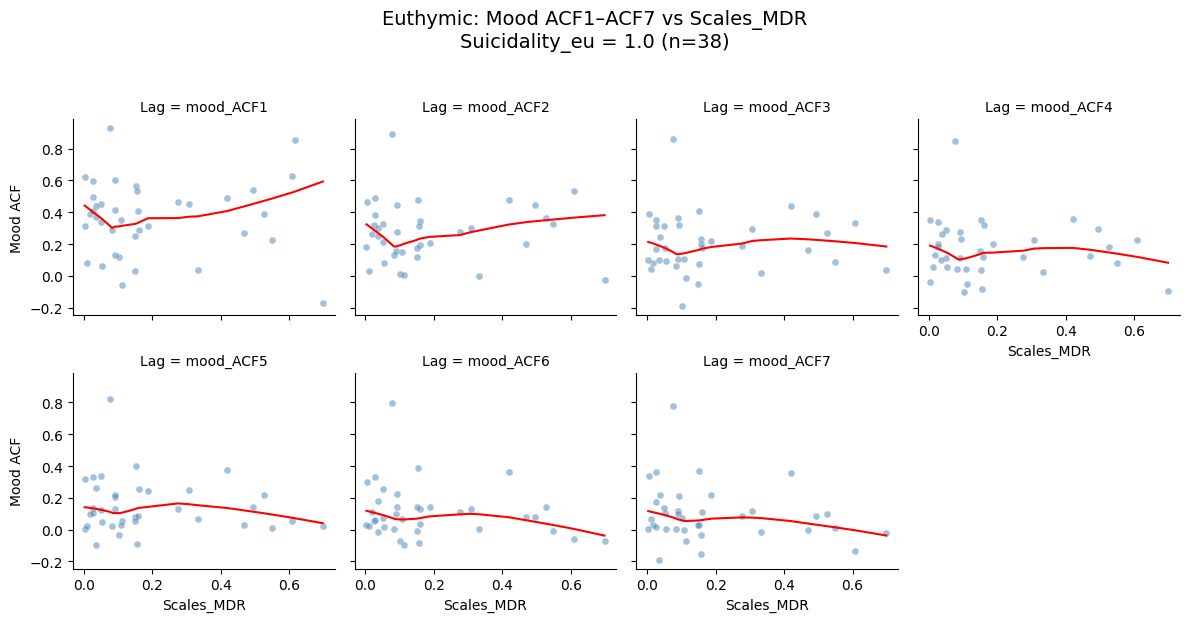


**Supplementary Figure 7.** Mood autocorrelation association with e-VAS missing data ratio (scales 1 to 7) for Suicide Group 2.


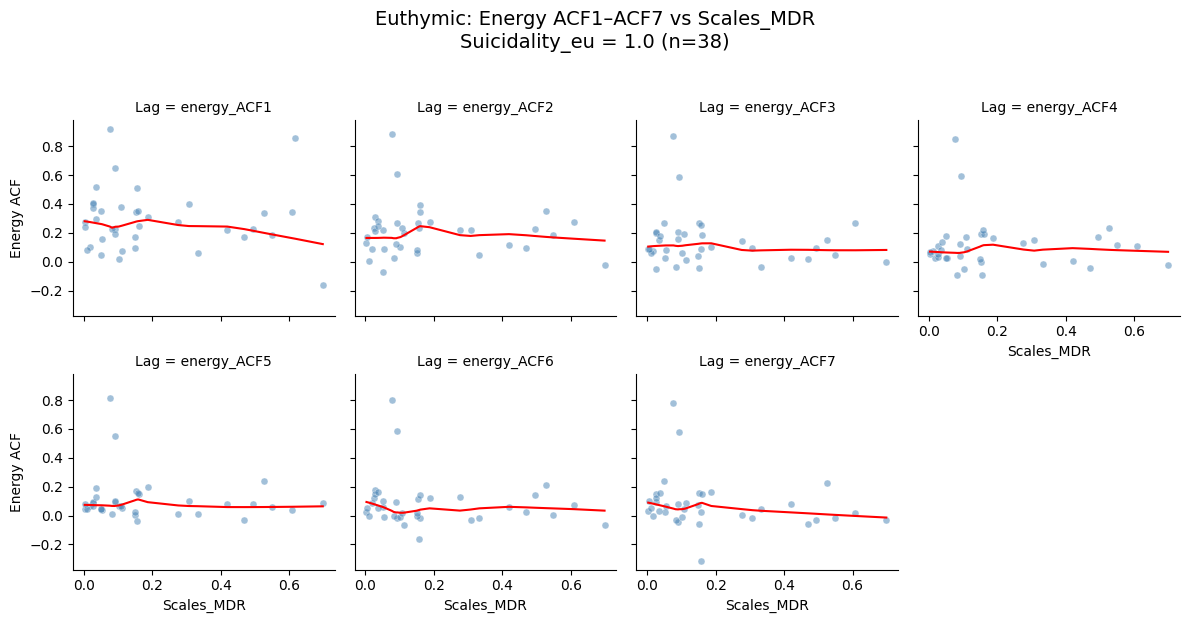


**Supplementary Figure 8.** Energy autocorrelation association with e-VAS missing data ratio (scales 1 to 7) for Suicide Group 2.


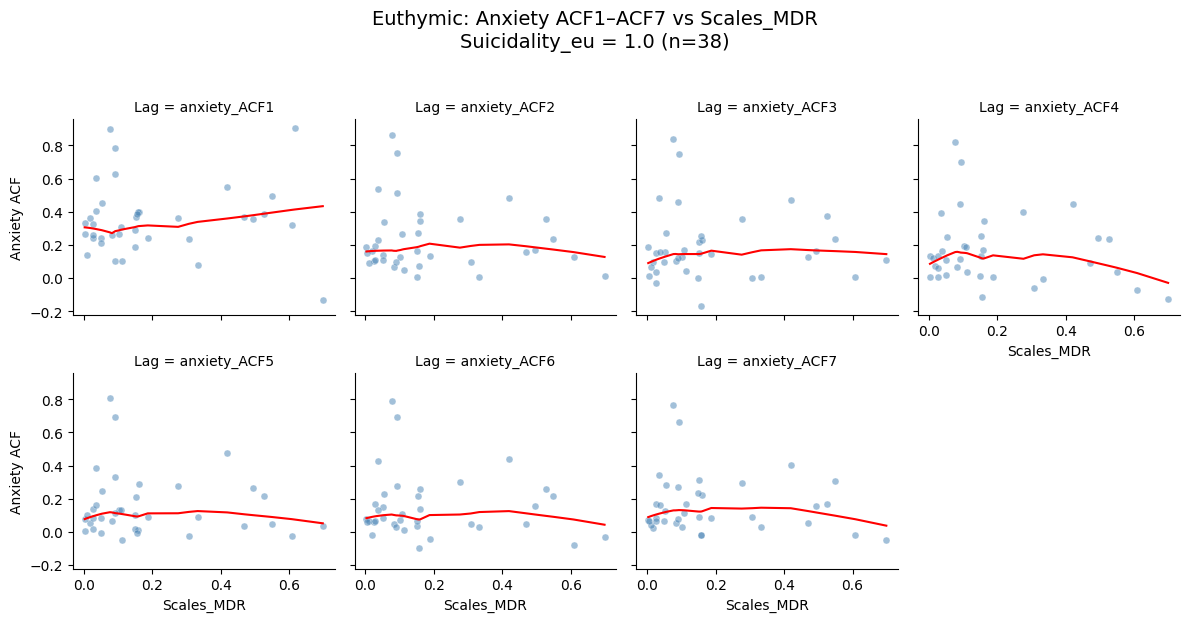


**Supplementary Figure 9.** Anxiety autocorrelation association with e-VAS missing data ratio (scales 1 to 7) for Suicide Group 2.


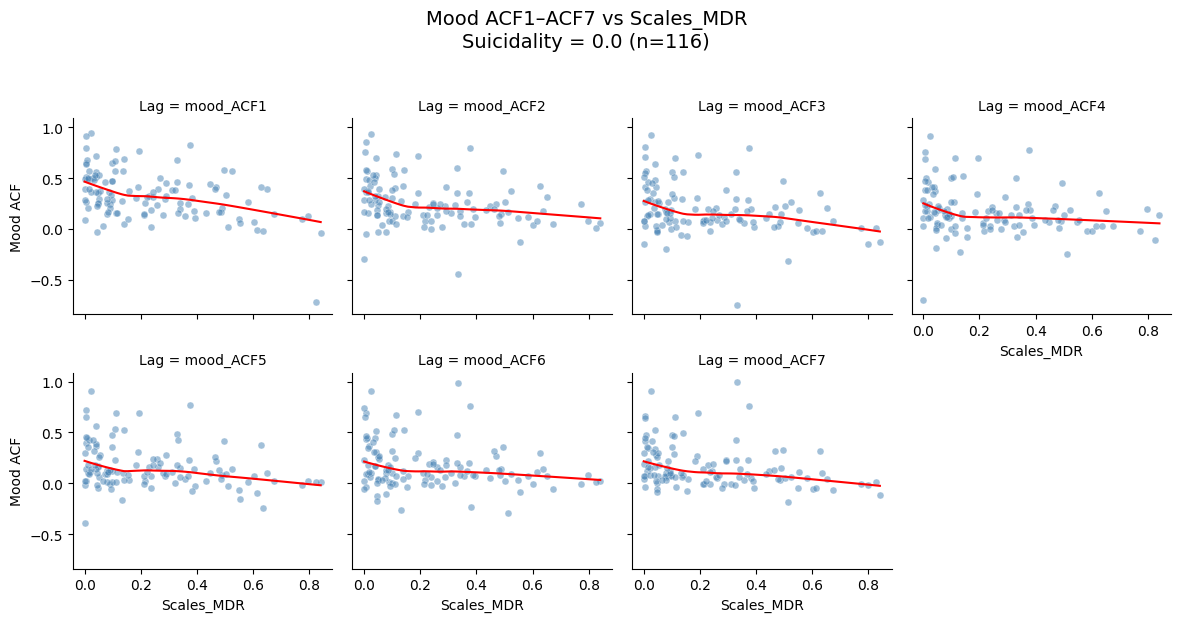


**Supplementary Figure 10.** Mood autocorrelation association with e-VAS missing data ratio (scales 1 to 7) for Suicide Group 3.


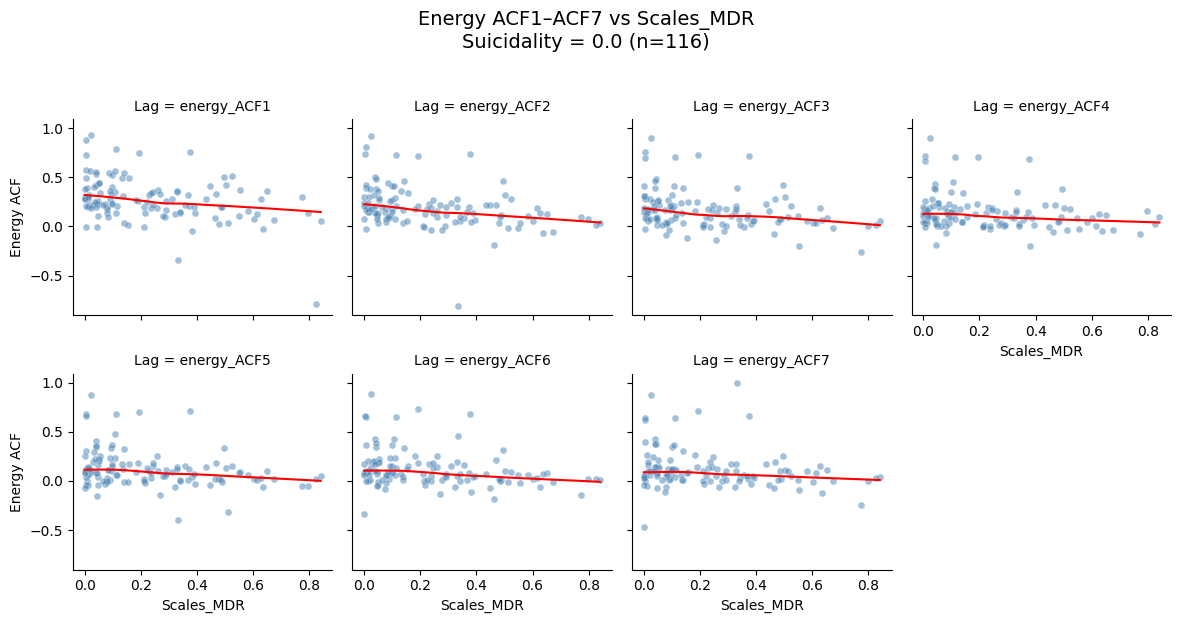


**Supplementary Figure 11.** Energy autocorrelation association with e-VAS missing data ratio (scales 1 to 7) for Suicide Group 3.


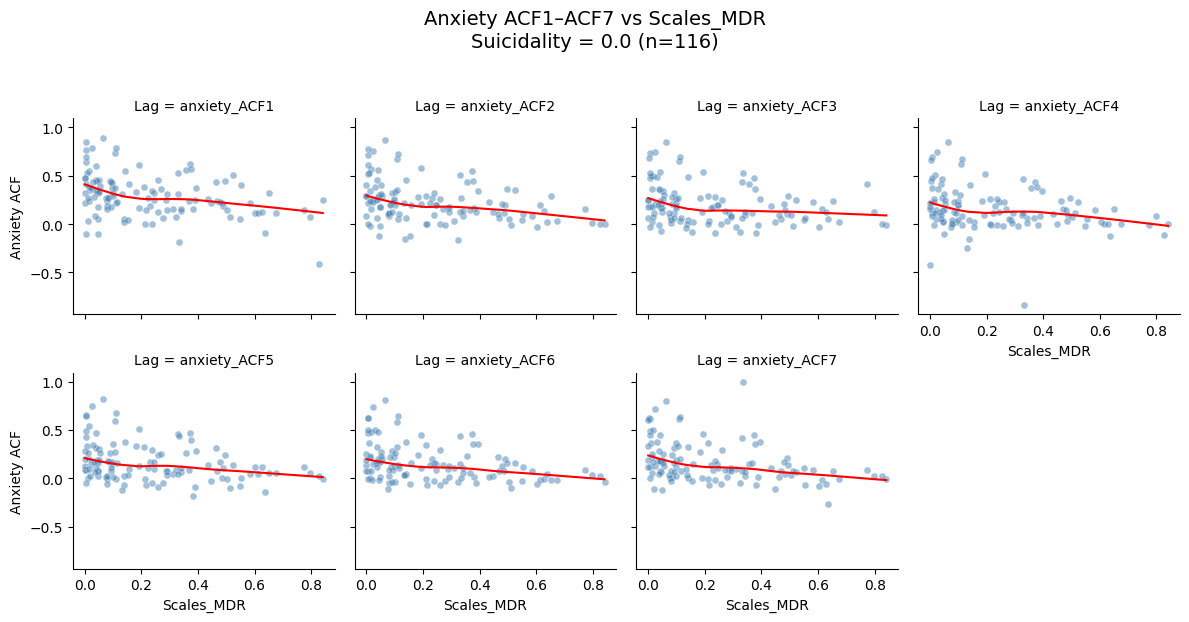


**Supplementary Figure 12.** Anxiety autocorrelation association with e-VAS missing data ratio (scales 1 to 7) for Suicide Group 3.

Longitudinal Missing Data Ratio Dynamics:


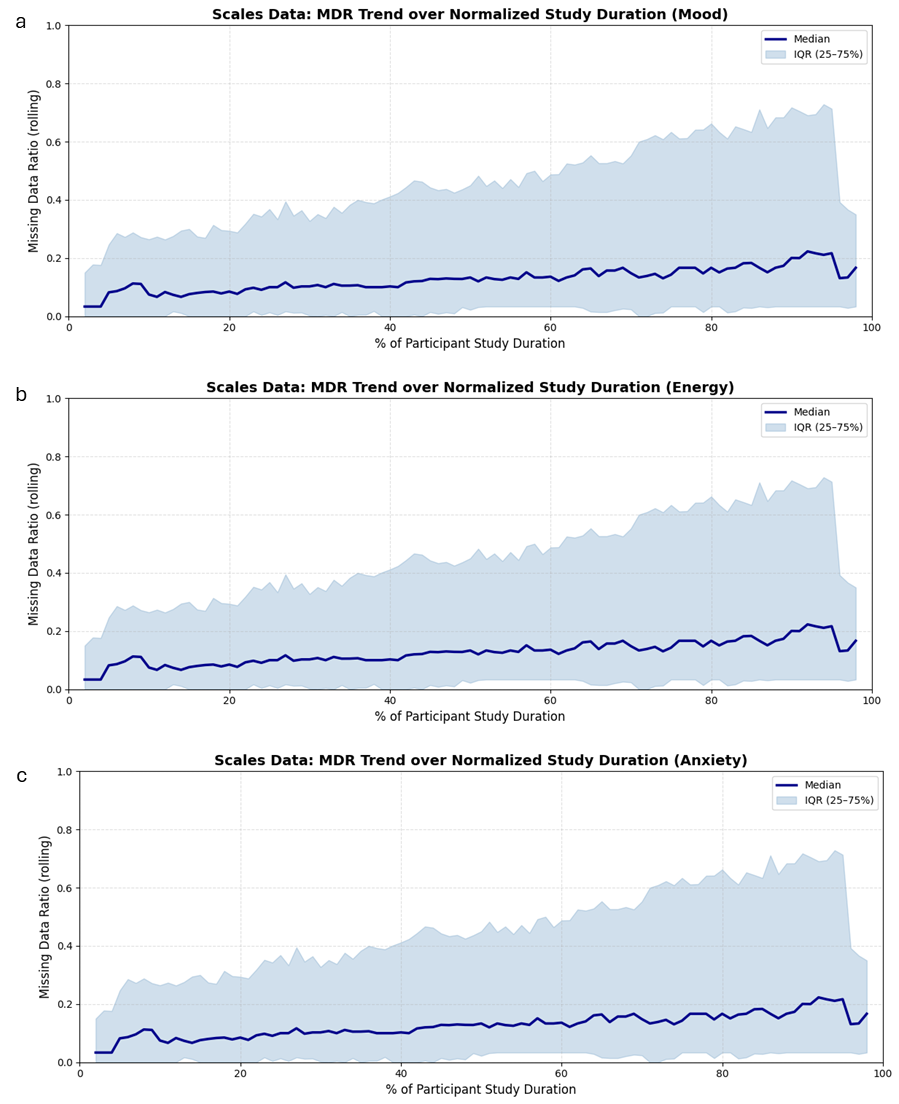


**Supplementary Figure 13.** Mood, energy, and anxiety missing data ratio longitudinal trends over normalized study course duration (solid blue line: median MDR per segment; shaded blue region: IQR (25-75%) confidence interval).


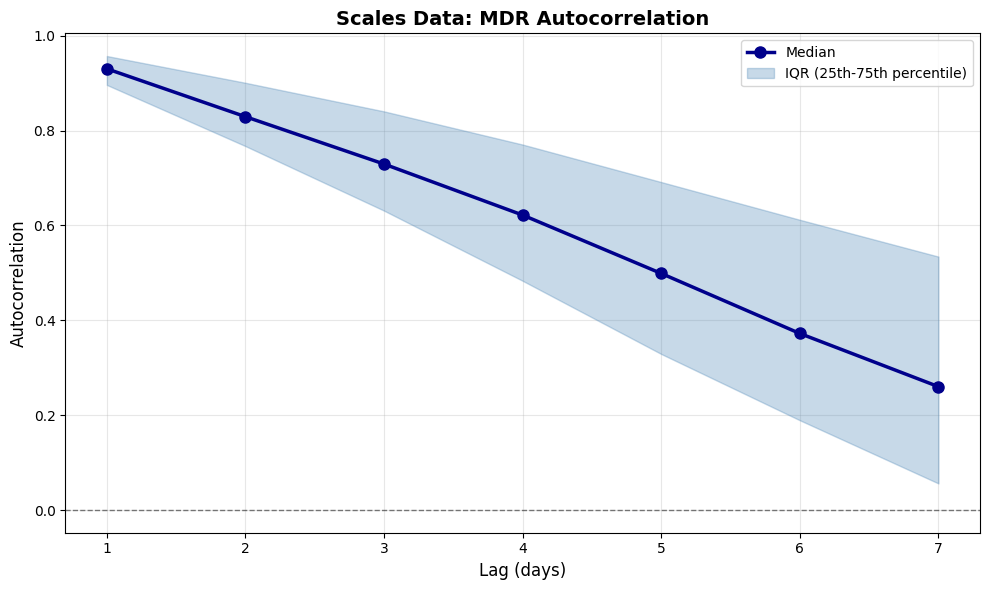


**Supplementary Figure 14.** Missing data ratio autocorrelation (lags 1 to 7) across participants (solid blue line: median ACF per lag; shaded blue region: IQR (25-75%) confidence interval).
